# Computational identification of HCV neutralizing antibodies with a common HCDR3 disulfide bond motif in the antibody repertoires of infected individuals

**DOI:** 10.1038/s41467-022-30865-9

**Published:** 2022-06-08

**Authors:** Nina G. Bozhanova, Andrew I. Flyak, Benjamin P. Brown, Stormy E. Ruiz, Jordan Salas, Semi Rho, Robin G. Bombardi, Luke Myers, Cinque Soto, Justin R. Bailey, James E. Crowe, Pamela J. Bjorkman, Jens Meiler

**Affiliations:** 1 Department of Chemistry, Vanderbilt University, Nashville, TN 37235 USA; 2 Center for Structural Biology, Vanderbilt University, Nashville, TN 37235 USA; 3 Division of Biology and Biological Engineering, California Institute of Technology, Pasadena, CA 91125 USA; 4 Department of Medicine, Johns Hopkins University School of Medicine, Baltimore, MD 21205 USA; 5 Vanderbilt Vaccine Center, Vanderbilt University Medical Center, Nashville, TN 37232 USA; 6 Department of Pediatrics, Vanderbilt University Medical Center, Nashville, TN 37232 USA; 7 Department of Pathology, Microbiology and Immunology, Vanderbilt University Medical Center, Nashville, TN 37232 USA; 8 Institute for Drug Discovery, Leipzig University Medical School, Leipzig, SAC 04103 Germany

**Keywords:** Protein function predictions, Hepatitis C, Molecular modelling, Antibodies

## Abstract

Despite recent success in hepatitis C virus (HCV) treatment using antivirals, an HCV vaccine is still needed to prevent reinfections in treated patients, to avert the emergence of drug-resistant strains, and to provide protection for people with no access to the antiviral therapeutics. The early production of broadly neutralizing antibodies (bNAbs) associates with HCV clearance. Several potent bNAbs bind a conserved HCV glycoprotein E2 epitope using an unusual heavy chain complementarity determining region 3 (HCDR3) containing an intra-loop disulfide bond. Isolation of additional structurally-homologous bNAbs would facilitate the recognition of key determinants of such bNAbs and guide rational vaccine design. Here we report the identification of new antibodies containing an HCDR3 disulfide bond motif using computational screening with the Rosetta software. Using the newly-discovered and already-known members of this antibody family, we review the required HCDR3 amino acid composition and propose determinants for the bent versus straight HCDR3 loop conformation observed in these antibodies.

## Abstract (web summary)

Identifying determinants of broadly neutralizing antibodies against hepatitis C virus (HCV) may guide HCV vaccine design. Here, the authors discover new anti-HCV antibodies using computational screening and analyze the amino acid composition and sequence-structure relationships in this antibody family.

## Introduction

Hepatitis C virus (HCV) resisted drug development efforts for a very long time, but recent progress in the development of direct-acting antivirals (DAAs) caused a dramatic change in HCV treatment strategies^1^. However, complete eradication of HCV remains an elusive goal. It has been estimated that ~50% of HCV-infected individuals in the US and more than 95% of the world’s HCV-infected populations are unaware of their HCV infection status and thus do not seek treatment^2,3^. The low infection awareness, the high cost of new therapies (making them unavailable in many parts of the world)^4^, and the lack of protection from HCV reinfection suggest that the development of a protective vaccine will be crucial for the global control of HCV infections. Moreover, drug resistance to each of the licensed DAAs has already been reported^1^. The impact of these resistant viruses on HCV treatment success at the population level is currently hard to predict.

It has been previously shown that the early production of broadly neutralizing antibodies (bNAbs) during HCV infection is associated with spontaneous HCV clearance^5–7^. Combinations of bNAbs targeting specific conserved epitopes on the surface of the HCV glycoprotein E2 increase neutralization breadth^8^. One of these highlighted epitopes is the front layer and the CD81 binding loop of E2, also commonly referred as an antigenic region 3 (AR3)^9^. It is the main target for multiple bNAbs. However, many of these bNAb epitopes overlap with the epitopes of other relatively narrow-breadth NAbs^10^. This fact complicates vaccine design efforts, since an effective anti-HCV vaccine likely needs to induce one or a combination of multiple specific bNAbs while avoiding an antibody response to non-neutralizing epitopes that may compete for binding with the desired bNAbs.

This vaccine design challenge can be addressed with the help of rapidly emerging epitope-focused vaccine design strategies^11–13^. Such designs can be sped up by a better understanding of the bNAb-antigen interactions at the molecular level. Atomic-level models of bNAb-antigen complexes obtained by X-ray crystallography or single-particle cryo-electron microscopy techniques play a crucial role in this process. However, in many cases, a single antibody-antigen structure may not be sufficient for differentiating essential from nonessential structural or biophysical Ab features. Identification and characterization of additional target bNAbs can help to overcome this complication and facilitate epitope-focused vaccine design efforts.

Multiple AR3-specific bNAbs reported to date are derived from the *V*_*H*_*1-69* gene independent of their source of origin: chronically infected donors^14,15^, individuals who spontaneously cleared the infection recently^7,16^ or many years ago^17^, and those who cleared one infection but then became persistently infected with a different HCV strain^18^. Despite sharing multiple common features, the *V*_*H*_*1-69*-encoded anti-HCV bNAbs’ pool is not quite homogeneous. As nicely summarized in a recent study by Chen et al.^19^, these Abs differ in the angle of approach and their footprint on the antigen, and can further be divided into subgroups. One of these subgroups has a distinguishable structural feature: a β-hairpin-shaped heavy chain complementarity-determining region 3 (HCDR3) loop stabilized by a disulfide bond.

Here, we describe the identification and characterization of novel Abs sharing common genetic and structural features with this previously described *V*_*H*_*1-69*-derived HCV E2-specific subgroup of bNAbs containing HEPC3^16^, HEPC74^16^, AR3C^14,20^, AR3A^14,21^, AR3X^22^, and HC11^15,23^. The new Abs, which cannot be discovered using conventional sequence-based search approaches, were found in the Ab repertoire of a previously infected individual using computational screening with the Rosetta software suite^24^. The characterization of these Abs allows for a more precise designation of this class of bNAbs and improvement of our understanding of the sequence-structure relationships of conformationally restricted long (e.g., HCDR3) loops.

## Results

### Search for structurally similar bNAbs

In our search for a promising family of anti-HCV bNAbs, we focused our attention on six *V*_*H*_*1-69*-derived bNAbs: HEPC3^16^, HEPC74^16^, AR3C^14,20^, AR3A^14,21^, AR3X^22^, and HC11^15,23^. Despite originating from four different individuals, these bNAbs are remarkably similar. They share a common heavy chain gene segment (*V*_*H*_*1-69*) and contain a D gene segment that encodes two cysteines in the HCDR3 loop^25^. The independent occurrence of such a combination of features in bNAbs from different people suggests that such bNAbs can be readily created from germline repertoires and that the epitope recognized by these bNAbs is an attractive and promising target for vaccine design. Moreover, the structures of two of these bNAbs, HEPC3 and AR3C, in complex with the antigen^20,25^ were known at the time of the project onset, allowing us to execute both sequence- and structure-based approaches to identify siblings of these Abs and define the structural determinants of this Ab family activity.

To focus our search, we obtained two libraries of heavy chain sequences from two individuals (subjects C110 and C117) with a history of spontaneous clearance of the HCV infection and from whom bNAbs HEPC74 and HEPC3, respectively, were isolated earlier^16^. These libraries were additionally filtered for sequences encoded by the *V*_*H*_*1-69* gene segment.

**Fig. 1:**
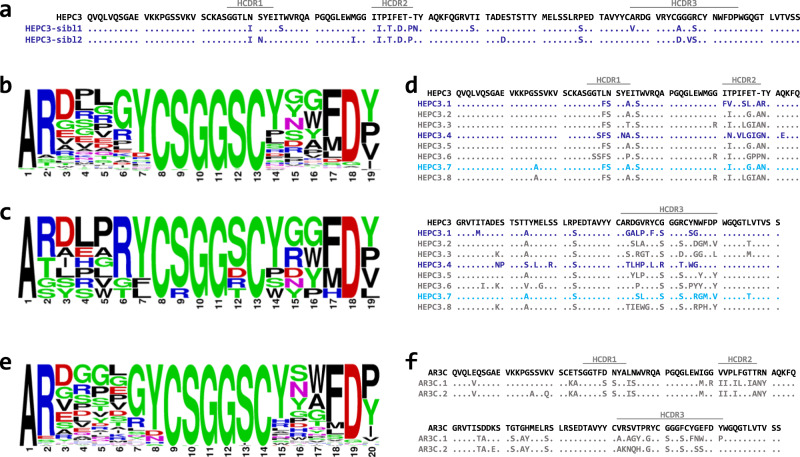
Analysis of HCDR3 loop diversity in the obtained heavy chains sequences library and sequences of the Abs selected for testing. **a**, **d**, **f** Alignments of the amino acid sequences of the heavy chain variable regions of **a** HEPC3 with its sibling Abs, **d** HEPC3 with selected by the P3SM approach HEPC3-like Abs, and **f** AR3C with selected by the P3SM approach AR3C-like Abs. Dots indicate the same amino acid as in the reference Ab sequence. Positions of the HCDR loops (IMGT definition) are marked above the alignment. **b**, **c**, **e** Amino acid frequency distribution in **b** all found HEPC3-like, **c** 8 selected by the P3SM approach HEPC3-like, and **e** all found AR3C-like HCDR3 loops. Amino acid frequency distributions were analyzed and visualized using WebLogo (https://weblogo.berkeley.edu/logo.cgi) web server.

Conventional sequence-based search for sibling Abs (sequences encoded by the same pair of V- and J-genes and showing at least 75% identity in the HCDR3 loop) yielded only two HEPC3-like Abs (designated HEPC3-sibl1 and HEPC3-sibl2) (Fig. 1a) from the subject C117 antibody library. No sibling Abs were found in the second library. Moreover, the pairwise similarity between six chosen AR3-specific bNAbs in most cases is lower than a traditionally used 75–80% identity cutoff for HCDR3 loops in sibling searches (Supplementary Fig. 1), meaning that a different search method might be required. Thus, we decided to use the homology modeling-based Position-Specific Structure Scoring Matrix (P3SM) approach to prioritize sequences for testing^26–28^.

Briefly, the P3SM approach was designed for the rapid, sequence similarity agnostic search of structurally and functionally homologous antibodies in sequence databases based on known Ab-antigen co-crystal structures. The protocol includes homology modeling of a subset of sequences using Rosetta^24^, building a scoring matrix, and subsequently applying this scoring matrix to assess sequences of interest. The P3SM approach has been applied successfully to a range of Abs, including the anti-HIV bNAb PG9^26^, the influenza hemagglutinin-specific Ab CH65^27^, and the anti-Marburg virus Ab MR78^28^.

In the current implementation, the P3SM method requires an exact length match between the surveyed regions of the template Ab and analyzed sequences. Both HEPC3 and AR3C bNAbs recognize their E2 epitope primarily through their HCDR3 loops^25^. This criterion restricted our sequence pool to only those sequences with 19 or 20 amino acid residue-long HCDR3s, matching the HCDR3 length of HEPC3 or AR3C, respectively. The D gene-encoded C-X-G-G-X-C amino acid motif in the HCDR3 of the HEPC3 and AR3C bNAbs allows the formation of a β-hairpin and decreases the conformational freedom of the amino acids in the tip of the HCDR3 loop, which was shown to play a critical role in HCV E2 recognition by this subgroup of neutralizing Abs^25^. Although the P3SM approach allows the scoring of potentially millions of sequences rapidly, there are limitations. In particular, this method excludes an exhaustive conformational sampling of the loops during the homology modeling stage. As a result, the prediction assesses the possibility of the given sequence to adopt the given conformation and form the required interactions. This approach does not guarantee that this conformation and the associated interactions will be the most favorable for the given sequence. Taking the above limitation into account, to minimize the number of false-positive predictions, we focused only on those sequences that already have a similarly pre-positioned C-X-G-G-X-C motif in their HCDR3 loops (in positions 8–13 for 19-amino acid long loops, and positions 9–14 for 20-amino acid long loops).

For the training dataset, we collected naturally occurring HCDR3s fulfilling the above-described criteria from a large Ab gene library obtained by next-generation sequencing (NGS) from healthy donors^29^. For each of the two template Abs (HEPC3 and AR3C), 1200 sequences were randomly selected, modeled, and analyzed to build the scoring matrix (see “Methods” for details). Application of the same search criteria (HCDR3 loop length and position of the C-X-G-G-X-C motif) to sequences obtained from two individuals with a history of spontaneous clearance of the HCV infection resulted in a relatively small candidate Ab sequence pool: 124 sequences with 19 amino acid-long HCDR3 loops (excluding the two already found during the sequence-based sibling search) and 71 sequences with 20 amino acid-long HCDR3 loops. Guided by the P3SM, we prioritized 35 19-residue HCDR3 loops and 18 20-residue HCDR3 loops from the above-described candidate sequence pool for detailed in silico analysis. We repeated homology modeling and manually evaluated the models. The evaluation was aimed to assess the probability of each sequence to adopt the conformation obtained by homology modeling. In particular, we filtered out models containing residues with unfavorable backbone conformations, unsatisfied hydrogen bonds, unrealistic predicted hydrogen bond networks, amino acids with high conformational flexibility in regions that needs to be rigid, etc. Following sequence selection with P3SM and manual review of the predicted structures, eight HEPC3-like (four from the subject C110 and four from the subject C117 sequence pools) and two AR3C-like HCDR3 sequences (all from the subject C117 sequence pool) were selected for further experimental validation (Fig. 1d).

We first compared the HCDR3 sequence profiles of all found (Fig. 1b) and eight selected (Fig. 1c) HEPC3-like 19-residue loops. While some positions appear to be tolerant to a diverse set of amino acids, the P3SM search prioritized certain amino acid residues in a number of locations. As was previously noted, a positively charged amino acid at position six (residue 98 based on Kabat numbering) seems to be favorable for this mode of E2 binding^25^. Interestingly, contrary to the previous observation, our results suggested that the next position might be occupied not only by a tyrosine but by another aromatic amino acid (phenylalanine) or by leucine. The residue after the first cysteine is glycine in both template bNAbs, most likely due to a somatic mutation of the D gene-encoded serine at that position. Our selected HCDR3 loop library was heavily enriched by sequences containing germline-encoded serine residues at that position.

The sequence profile of all AR3C-like 20-residue HCDR3 loops (Fig. 1e) is similar to the one of all 19-residue loops. However, none of the individual amino acid combinations scored particularly well. Based on the overall analysis, we chose to test two variants, although neither had a positively charged amino acid residue at position seven (Fig. 1f).

### Seven out of twenty-two tested mAbs bound HCV E2 ectodomains

For each of the ten HCDR3 sequences selected by our P3SM approach, we generated two recombinant Abs. The first one, termed a chimeric Ab, recapitulated the computationally tested proteins: the HCDR3 loop was placed on the appropriate Ab framework (HEPC3 for 19-residue HCDR3 loops [designated HEPC3.1_HCDR3_ to HEPC3.8_HCDR3_] and AR3C for 20-residue HCDR3 loops [designated AR3C.1_HCDR3_ and AR3C.2_HCDR3_]). The second, termed native Ab, consisted of the full heavy chain variable region sequence (Fig. 1d, f) from which the HCDR3 had been extracted previously for analysis (designated HEPC3.1-8 or AR3C.1-2). The two sibling Abs were tested only as full-length sequences. In all cases, the recombinant Abs were expressed with the corresponding light chain from either HEPC3 (HEPC3-V_L_) or AR3C (AR3C-V_L_) due to the lack of information about the native light chain pairing for these sequences. However, while in both parental Abs the light chain shows no direct interaction with the antigen, we hypothesized that such pairings have a high probability of being tolerated.

**Fig. 2:**
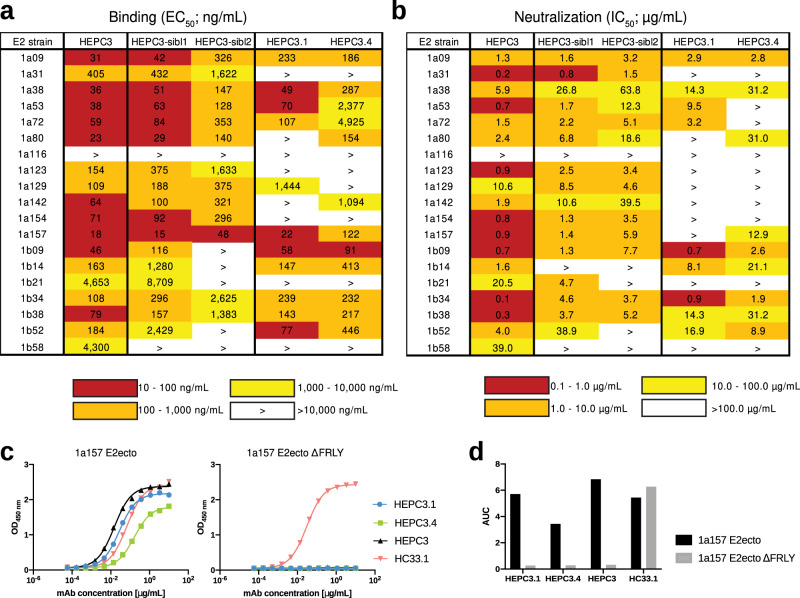
HEPC3.1, HEPC3.4, and sibling HEPC3 Abs display broad binding activity and neutralize multiple HCV strains. **a** Heat map showing the binding of Abs to a panel of HCV genotype 1 E2 glycoproteins. The EC_50_ value for each E2-Ab combination is shown, with dark red, orange, yellow, or white shading indicating high, intermediate, low, or no detectable binding, respectively. The “>” symbol indicates EC_50_ values >10 µg/mL or combinations were OD_450_ values at the highest antibody concentration tested were lower than 0.5. One experiment representative of two independent experiments is shown. **b** Heat map showing neutralization activities of Abs measured using a panel of genotype 1 HCVpp. The IC_50_ value for each E2-Ab combination is shown, with dark red, orange, yellow, or white shading indicating high, intermediate, low, or no detectable neutralization activity, respectively. The “>” symbol indicates IC_50_ values >100 µg/mL or combinations in which percent neutralization at the highest antibody concentration tested was lower than 50%. **c** Binding of HEPC3.1, HEPC3.4, and reference bNAbs to a 1a157 E2 glycoprotein ectodomain (1a157 E2ecto) and 1a157 E2 variant that contains alanine substitutions that disable binding of HEPC3-like bNAbs that target AR3 antigenic site (1a157 E2ecto ΔFRLY). Introduced alanine substitutions in 1a157 E2ecto ΔFRLY do not disrupt the binding of HC33.1-like bNAbs targeting the antigenic site 412 (AS412) that lies in close proximity to the E2 front layer. One experiment representative of two independent experiments is shown. Source data are provided in the Source Data file. **d** Binding data from (**c**) is shown as the area under the curve (AUC).

We tested all 22 resulting Abs using a panel of E2 ectodomains from genotype 1 HCV strains. Both sibling Abs and five Abs derived from three HCDR3 sequences prioritized by P3SM (two pairs (native mAb/chimeric mAb) and one additional chimeric Ab) bound to HCV E2 ectodomains in ELISA (Fig. 2a, Supplementary Figs. 2,  3). Interestingly, in cases where both native and chimeric Abs showed binding (HEPC3.1**/**HEPC3.1_HCDR3_ and HEPC3.4**/**HEPC3.4_HCDR3_), the native Abs displayed stronger binding and broader reactivity to E2 proteins than the chimeric Abs (Supplementary Fig. 2). Mutations outside the HCDR3 loop introduced during the affinity maturation of these native Abs may have been selected for different reasons, including increasing the overall protein stability, affinity to other targets or to eliminate self-reactivity, among others. However, there is also a possibility that these Abs were indeed elicited in response to HCV infection and contain additional mutations that facilitate correct placement of these HCDR3 loops and/or shape other regions for interaction with the antigen. Moreover, both HEPC3.1 and HEPC3.4 originated from the subject C110 sequence pool but were discovered using the bNAb HEPC3, isolated from the subject C117, as a search template, confirming that the P3SM approach can successfully identify Abs that evolved independently for binding the same epitope.

To assess the probability of finding functional E2 ectodomain-binding Abs in the Ab repertoires of patients who spontaneously cleared HCV infection just by chance, we also tested ten randomly selected C-X-G-G-X-C HCDR3 motif-containing Abs (Supplementary Fig. 4). None of these ten Abs recognized E2 antigen in ELISA (Supplementary Fig. 5).

Among the characterized Abs, HEPC3-sibl1 demonstrated the highest breadth: of all tested HCV genotype 1 E2 glycoproteins, this Ab fails to recognize only one that is recognized by bNAb HEPC3. However, the strength of binding to some strains is reduced. The other three Abs showed overall similar breadth, with HEPC3.1 characterized by tight binding (EC_50_ < 100 ng/mL) to more variants (Fig. 2a).

### Newly identified Abs neutralize multiple HCV strains

We further assessed HCV neutralizing potency and breadth of the HEPC3-sibl1, HEPC3-sibl2, HEPC3.1, and HEPC3.4 mAbs using a panel of genotype 1–6 HCV pseudoparticles (HCVpp). In accordance with the binding data (Fig. 2a), all discovered Abs neutralized multiple HCV strains (Fig. 2b, Supplementary Fig. 6). Both sibling Abs demonstrated similar breadth and potency; however, they are less broad and potent than HEPC3. HEPC3.1 and HEPC3.4 mAbs were able to neutralize a smaller number of tested HCV strains. Notably, the highest (for HEPC3.1) and the second-highest (for HEPC3.4) neutralization activity was detected against 1b09 strain HCVpp. An E2 ectodomain from this particular HCV strain, previously co-crystallized in complex with HEPC3 Ab^25^, was used as an input for the P3SM search in this work that resulted in the discovery of HEPC3.1 and HEPC3.4 Abs.

Both HEPC3-sibl1 and HEPC3-sibl2 HCDR3 loop sequences differ from the bNAb HEPC3 sequence in only three positions, including two and three mutations, correspondingly, in a highly structurally restricted region between two cysteines at the tip of the loop. Thus, the observed similarity to HEPC3 in binding and neutralization activity was not unexpected. In HEPC3.1 and HEPC3.4, on the other hand, almost half of the HCDR3 loop sequence (8 and 9 positions out of 19, respectively) is altered. We further characterized the latter two Abs as their higher sequence diversity should provide more insights into the structural and/or biophysical features required for AR3 antigenic site recognition.

### HEPC3.1 and HEPC3.4 likely target the AR3 antigenic site similarly to the HEPC3 bNAb

We investigated whether the computationally identified mAbs HEPC3.1 and HEPC3.4 bind to the AR3 antigenic site similar to the template Abs by using an ELISA binding assay. For this assessment, we used a 1a157 E2 knockout variant that contains alanine substitutions that disable the binding of HEPC3-like bNAbs targeting the AR3 antigenic site (1a157 E2ecto ΔFRLY). As controls, we used the AR3-specific HEPC3 bNAb and the HC33.1^30^ bNAb that binds to the antigenic site 412 (AS412) that lies in close proximity to the E2 front layer. As expected, in contrast to HC33.1 bNAb that bound equally well to 1a157 E2 ectodomain and 1a157 E2ecto ΔFRLY, both HEPC3.1 and HEPC3.4 failed to bind to 1a157 E2ecto ΔFRLY, suggesting that the new Abs target the AR3 antigenic site similarly to HEPC3 bNAb (Fig. 2c, d).

### Crystal structures of HEPC3.1 and HEPC3.4 Fabs reveal bent HCDR3 loop conformation

All six previously characterized AR3-specific Abs from the *V*_*H*_*1-69*-derived, HCDR3 disulfide bond motif-containing Ab family that were co-crystallized with the antigen (HEPC3, HEPC74, AR3A, AR3C, AR3X, and HC11) demonstrate the same pattern of antigen recognition: the interaction is driven primarily by HCDR3 loop contacts as assessed both by the predicted interaction energy and the calculated buried surface area; the only exception is AR3X with its atypically long HCDR2 loop (Supplementary Fig. 7**)**. However, these Abs can be structurally organized into two distinct subgroups based on the HCDR3 loop preference in adopting either straight (HEPC3, AR3X, and HEPC74) or bent (AR3C, AR3A, and HC11) conformations. The shape of the HCDR3 loop alters how the Ab approaches the antigen.

**Fig. 3:**
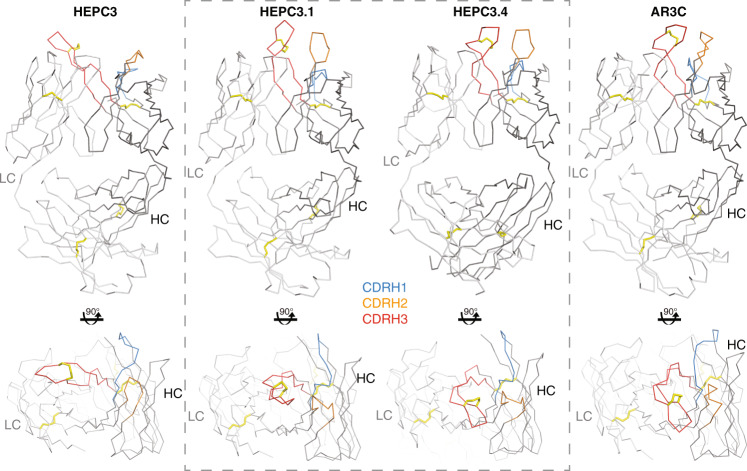
The HCDR3 loop in HEPC3.1 and HEPC3.4 Abs adopts a bent orientation. Side (top) and top (bottom) views of Fab apo structures of HEPC3 (PDB ID: 6MED), HEPC3.1 (this study), HEPC3.4 (this study), and AR3C (PDB ID: 6MEF). The crystal structures were superimposed on their V_H_ domains. Protein backbones are shown as ribbons, and CDR loops are blue (HCDR1), orange (HCDR2), and red (HCDR3). Disulfide bonds are shown as yellow sticks.

To explore structural features of the discovered Abs, crystal structures of the antigen-binding fragments (Fabs) of HEPC3.1 and HEPC3.4 were determined to resolutions of 2.8 and 3.3 Å, respectively. Contrary to our expectations, both structures revealed a bent HCDR3 conformation like the one previously found in the AR3C, AR3A, and HC11 bNAbs, rather than an extended (straight) loop as seen in the HEPC3 mAb that was used as a template for this P3SM search (Fig. 3, Supplementary Fig. 8).

**Fig. 4:**
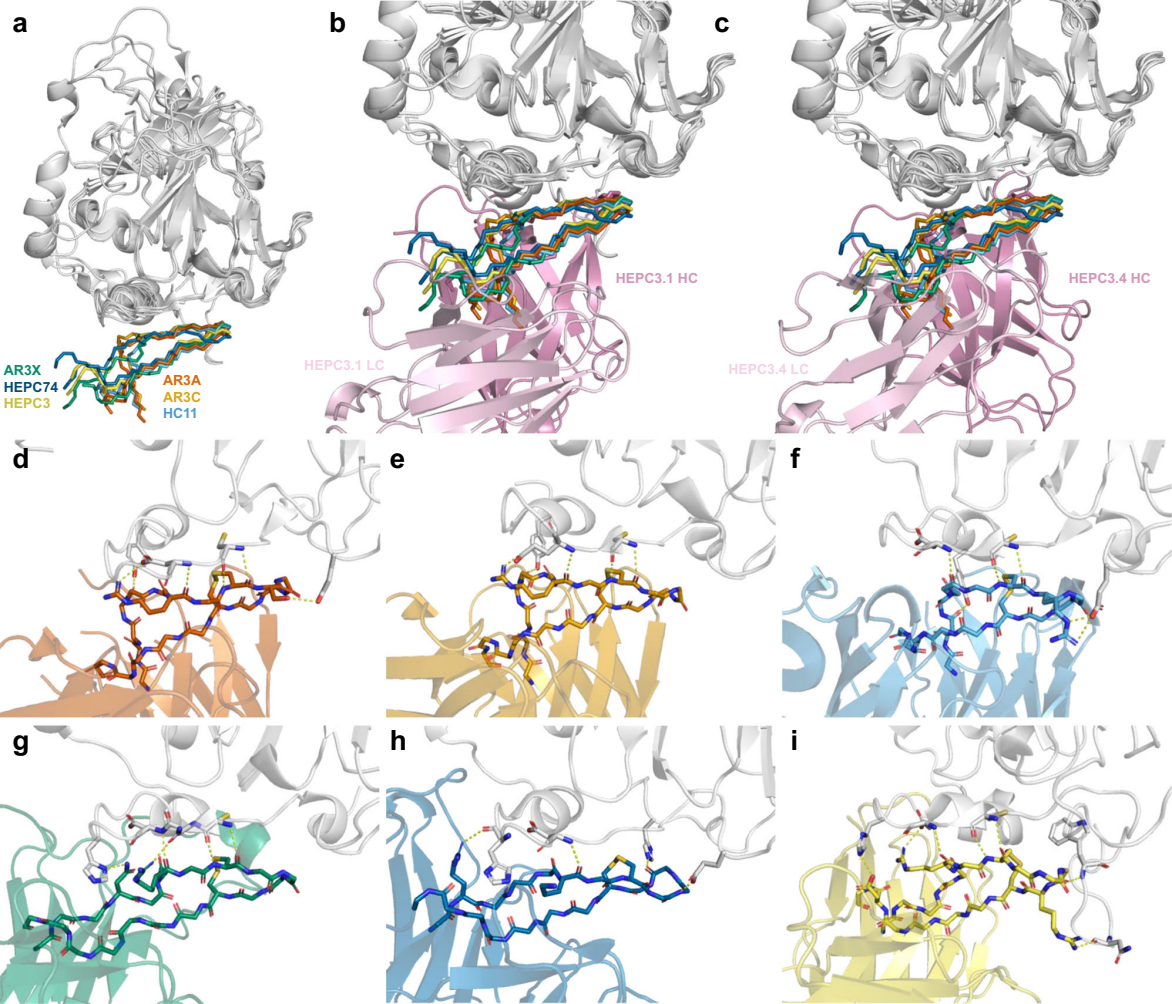
Interactions of the HCV neutralizing *V*_*H*_*1-69* gene-encoded Abs encompassing a common HCDR3 disulfide bond motif with the front layer of HCV glycoprotein E2. **a** All six Abs of the described family demonstrate the same pattern of antigen recognition mediated by their HCDR3 loop. Shown is the overlay of the HCV glycoproteins E2 from the co-crystal structures with bNAbs HEPC3, HEPC74, AR3A, AR3C, AR3X, and HC11. The glycoproteins are shown as a gray cartoon. The backbones of the HCDR3 loops of the Abs are shown as sticks. **b**, **c** The HCDR3 loop tips of the unliganded HEPC3.1 (**b**) and HEPC3.4 (**c**) Fabs were aligned with the observed common antigen recognition pattern of the *V*_*H*_*1-69* gene-encoded Abs with an HCDR3 disulfide bond motif. The absence of major clashes suggests that HEPC3.1 and HEPC3.4 Abs might interact with the antigen in the same way as other front layer-specific bNAbs. **d**–**i** Polar interactions between HCV glycoprotein E2 and HCDR3 loops of **d** AR3A, **e** AR3C, **f** HC11, **g** AR3X, **h** HEPC74, and **i** HEPC3 Abs, as seen in the crystal structures.

The tip of the HCDR3 loop in previously characterized *V*_*H*_*1-69*-derived, HCDR3 disulfide bond motif-containing Abs forms a β-hairpin that interacts with an epitope in the front layer of E2 (Fig. 4a). An overlay of the HEPC3.1 and HEPC3.4 Fab HCDR3 loop tips with known Ab co-crystal structures does not reveal significant clashes (Fig. 4b, c). This observation, as well as the absence of binding to the front layer 1a157 E2 knockout variant (Fig. 2c, d), suggests that the P3SM-discovered HEPC3.1 and HEPC3.4 Abs interact with E2 in a similar way.

### Reevaluation of the HCDR3 loop motif of the AR3-specific Abs

The Ab HCDR3 loops interact with E2 mostly through backbone-backbone interactions of the β-hairpin at the tip of the loop. These interactions are supported by side chain–side chain and side chain–backbone interactions (Fig. 4d–i). The R/K-Y-C-G-G-G-X-C HCDR3 motif has been suggested to be important for E2 recognition^25^. While there is no doubt of the critical role of the two cysteines and two middle glycines (C-X-G-G-X-C motif) for formation of the observed β-hairpin, the residue after the first cysteine may not be required to be glycine, as observed in HC11, AR3A, AR3X, and the newly described HEPC3-sibl1, HEPC3-sibl2, HEPC3.1, and HEPC3.4. The positively charged amino acid in the −2 position from the first cysteine is not present in HC11, and the lysine residue in HEPC74 seems not to be involved in the interaction with the antigen. The residue preceding the first cysteine does not interact with the antigen and most likely participates in overall loop stabilization and positioning. Amino acids in addition to tyrosine are now also found in that position. Overall, the amino acid composition of the HCDR3 tip in this class of Abs appears to be less restricted than previously assumed.

### Proline and glycine in the HCDR3 loop may promote bent HCDR3 loop conformations

**Fig. 5:**
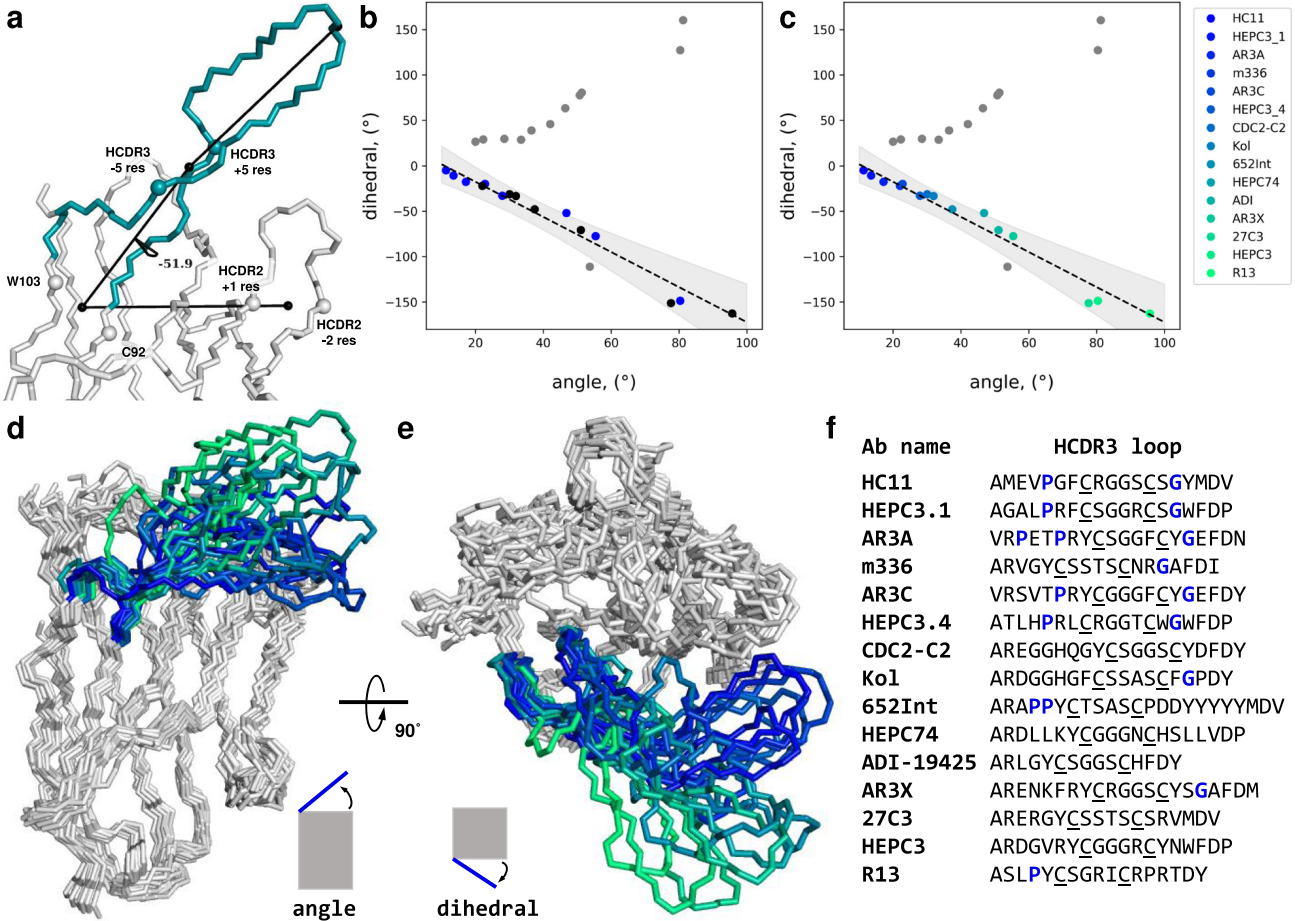
Analysis of the Ab structures with an HCDR3 loop CXXXXC motif. **a** The four points on an Ab that were used for the description of the overall shape of the HCDR3 loops in the dataset on the example of HEPC74 (PDB ID 6MEE). The HCDR3 loop is colored. The Cα atoms used for calculations are shown as spheres. The four calculated points are shown as black spheres and are connected by black lines. **b**, **c** Calculated angles and dihedrals for HCDR3 loops of all 27 found Abs. Source data are provided in the Source Data file. **b** The values for the HCV-specific Abs are shown as blue dots. The unrelated Abs that are following the same trend as the HCV-specific Abs are shown as black dots. Also shown are the estimated linear correlation curve (dashed line) for the proposed trend and +/−3σ uncertainty around the estimated curve (shadowed area). **c** The Abs that seem to follow the trend of the rigid body HCDR3 loop rotating away from the heavy chain and the Ab Fv region, in general, are color-coded based on the distance (blue—closer, green— further away). A side view (**d**) and a top view (**e**) on the aligned Fv regions of the Ab heavy chains. The HCDR3 loops are colored according to the legend on the panel (**c**). The ultra-long HCDR2 loop of AR3X is not shown. **f** Sequences of the HCDR3 loops (IMGT definition) of the Abs shown on the panels (**d**) and (**e**). The two cysteines in each HCDR3 loop are underscored. Prolines in the first half of the loop and glycines in the second half of the loop are colored blue.

To better understand possible structural determinants for the observed nearly bimodal separation of the HCDR3 loops of this class of anti-HCV Abs into straight and bent conformations, we searched the PDB database for similar Abs. Our search for human Abs determined with the resolution ≤ 3.0 Å with a CXXXXC motif roughly in the middle of the HCDR3 yielded 27 Abs with unique HCDR3 sequences (August 2020). Taking into account the relatively small number of these Abs combined with high variability in the observed length and shapes of the loops, it was apparent that clustering would be inefficient in this case. However, an additional conformational restriction enforced by the disulfide bond results in a relatively narrow, hairpin-like overall HCDR3 loop shape in the obtained dataset. Based on this observation we describe the overall shape of these HCDR3 loops by two parameters: (i) the proximity of the HCDR3 loop tip to the Ab heavy chain variable domain in general and (ii) the position of the HCDR3 loop tip relative to the heavy chain—light chain Ab plane. For this we specified four points: (1) the base of the HCDR3 loop (a midpoint between C_α_ of C92 and W103), (2) the base of the HCDR2 loop (defined here as a midpoint between C_α_ of the first and second to the last residues of the HCDR2), (3) the base of the HCDR3 loop head (non-torso region, defined here as a midpoint between C_α_ of the fifth and the fifth from the end residues of the HCDR3), and (4) the tip of the HCDR3 loop (defined here as a C_α_ atom of the middle HCDR3 loop residue in case of odd HCDR3 length and as a midpoint between C_α_ of the two middle residues of the HCDR3 in case the loop has even number of residues). We defined the first parameter as an angle between vectors $\overrightarrow{12}$ and $\overrightarrow{34}$. We defined the latter parameter as a dihedral angle between two planes specified by the four points described above (Fig. 5a).

Interestingly, more than half of the analyzed Abs, including all anti-HCV Abs in the dataset, seem to follow a trend where moving of the tip of the HCDR3 loop further from the Ab Fv region coincides with its moving away from the heavy chain toward the light chain (Fig. 5b). We confirmed this trend by visual inspection of that subset of Ab structures (Fig. 5c–e). The observed movement can be explained by the relative rigidification of the HCDR3 loop head stabilized by the disulfide bond, which enforces coordinated movement of the loop in response to changes in HCDR3 lower region. To investigate the cause of these HCDR3 loop head movements, we compared the HCDR3 sequences of these 15 Abs (Fig. 5f). At the top of the list, which corresponds to the structures with the most pronounced bent conformation of the HCDR3 loop, we found repetitive co-appearance of proline and glycine residues correspondingly just after the torso portion in the first half of the HCDR3s and before the start of the torso region in the second half. Both proline and glycine have unique backbone dihedral angle distributions compared to the other amino acids, causing them to disrupt secondary structures and promote loops and turns^31^. The observed combination of the proline, glycine, and disulfide bond is not mandatory for the bent conformation of the HCDR3 loop, as can be seen in the case of the m366 Ab in which the proline is missing^32^. While not seen in our relatively small dataset, the opposite situation, where in the presence of all three above-mentioned elements the HCDR3 loop remains straight conformation, is most likely also possible. However, this combination of features should be considered in Ab design projects as a possible way for promoting alteration of the conformation of long Ab loops.

### Conformational dynamics of HEPC3, HEPC3.1, and HEPC3.4 HCDR3 loops

Despite previous experimental evidence supporting HCDR3 loop bent conformations as effective interaction geometries for certain HCV AR3-specific Abs (AR3C, AR3A, and HC11), the long length of the HEPC3.1 and HEPC3.4 HCDR3 loops (19 amino acids) coupled with obtaining only their apo state crystals remain the possibility that presence of the bent conformation may be a crystallographic artifact. To test the hypothesis that HEPC3.1 and HEPC3.4, but not HEPC3 HCDR3 loops are more stable in the bent conformation than in the straight conformation, we performed explicit solvent molecular dynamics (MD) simulations of each Ab in the apo state.

**Fig. 6:**
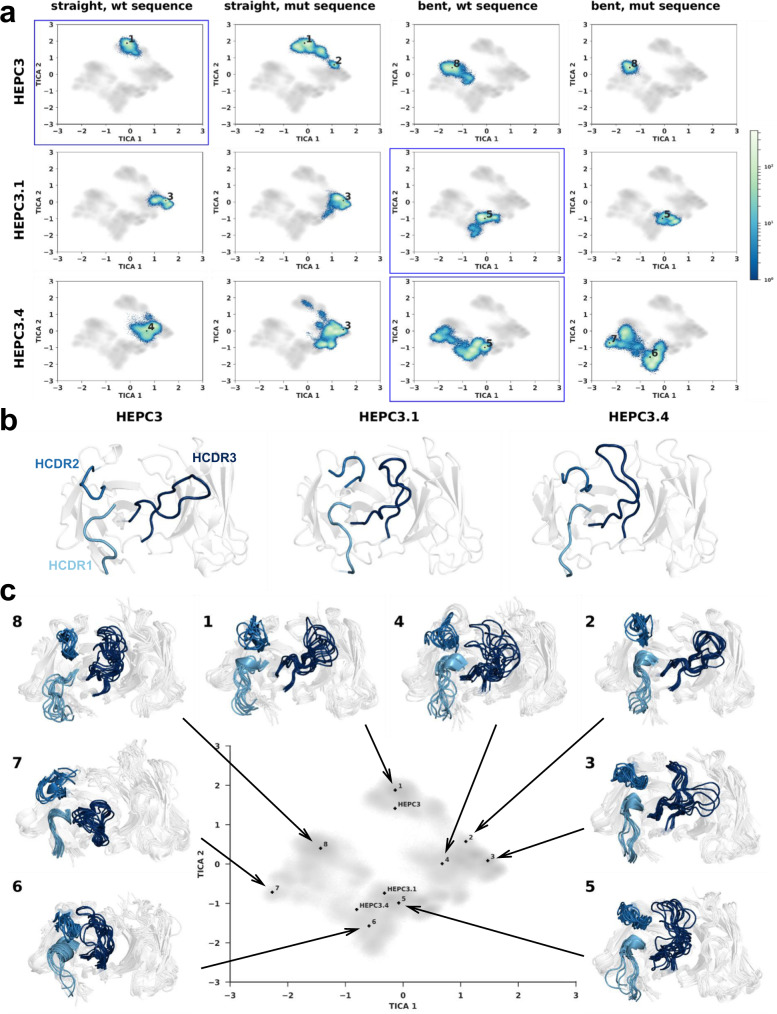
HCDR3 loop conformational dynamics. **a** 2D-histograms of individual Ab simulations projected onto the first two time-lagged independent components of the combined Ab TICA-transformed conformational space. Histograms corresponding to simulations initialized with the crystallographic conformations are outlined in blue. **b** Cartoon representation of the structures of the Abs HEPC3, HEPC3.1, and HEPC3.4 as observed in the crystal structures of the apo Fabs. HCDR1, HCDR2, and HCDR3 loops are colored light blue, blue, and dark blue, respectively. **c** Ensembles of 10 structures from representative conformational clusters. Structures are shown as cartoons. HCDR1, HCDR2, and HCDR3 loops are colored light blue, blue, and dark blue, respectively. Numbers in (**a**) and (**c**) correspond to minima sampled to display ensembles.

We conducted six independent 5.0 µs simulations of HEPC3, HEPC3.1, or HEPC3.4. Each Ab was simulated from two starting conformations: the HEPC3 crystallographic pose with the straight HCDR3 loop or the HCDR3.1/3.4 crystallographic pose with the bent HCDR3 loop. The non-crystallographic conformation of each Ab (e.g., bent conformation of HEPC3) was modeled with Rosetta using the other structures (see “Methods”). A total of 180 µs of MD simulations were performed for these Abs. We analyzed the trajectories by performing time-lagged independent component analysis (TICA) on a large set of intramolecular distances, angles, and dihedrals to identify kinetically associated high probability conformations (Fig. 6).

The investigated Abs displayed variable levels of flexibility in their HCDR3 loops with HEPC3 being the most rigid and HEPC3.4 being the most flexible. In support of our hypothesis, the conformational space sampled by HEPC3.1 and HEPC3.4 (Fig. 6c ensembles 3–5) is only sparsely populated with HEPC3-like straight HCDR3 loop conformations (Fig. 6c ensemble 1) independent of the starting conformation (Fig. 6a columns 1 and 3, Fig. 6b and c). Interestingly, the HEPC3 HCDR3 loop predominantly remained in the conformation (bent or straight) from which the simulation was initiated (Fig. 6c, clusters 1 and 8), suggesting it may have fewer energetically favorable conformational states. Taken together, our MD simulation results suggest that the intrinsic dynamics of the HCDR3 loops differ but crystallographic artifacts are unlikely to be responsible for the observed bent HCDR3 loop conformation in HEPC3.1 and HEPC3.4 Abs.

We also tested our hypothesis that proline and glycine substitutions at the base of the HCDR3 loop bias the loop toward a bent conformation. For this we performed an analogous set of simulations on variants of the three original Abs with mutations at positions 97 and 100 G (Kabat numbering): HEPC3-PG (HEPC3-V_97_P-N_100G_G), HEPC3.1-VN (HEPC3.1-P_97_V-G_100G_N), and HEPC3.4-VN (HEPC3.4-P_97_V-G_100G_N).

HEPC3-PG displayed increased conformational diversity when the simulations were started from the straight conformation (Fig. 6a row 1, columns 1 and 2). In contrast, simulations starting from the bent conformation displayed narrower conformational sampling (Fig. 6a row 1, columns 3 and 4). These results suggest that in the HEPC3 HCDR3 loop, proline and glycine may modulate the relative stability of the bent and straight conformations.

We then evaluated P_97_V and G_100G_N mutations, the native amino acids of the HEPC3 sequence, in HEPC3.1 and HEPC3.4 Abs. In both cases, we also observed changes in the sampling landscape of the mutant Abs compared to the wild type (Fig. 6a). For example, HEPC3.4-VN sampled HEPC3-like straight HCDR3 loop conformation more often. However, it was not the dominant conformation for this Ab. In other cases, the introduced mutations prompted the Ab to explore completely new areas of the conformational landscape. These simulations did not lead to any obvious alterations in conformational preferences of the HCDR3 loops, suggesting that they may be far from equilibrium despite extensive sampling. Altogether, our observations suggest that residue identity at positions 97 and 100 can impart substantial changes in rigidity/flexibility, but that the overall conformational preference is likely a broader topological property of HCDR3 loop or whole Ab sequence.

Finally, we analyzed the possible influence of other parts of the Ab on the HCDR3 conformation. The CDRH1 and CDRH2 sequences of HEPC3.1 and HEPC3.4 differ significantly from those of HEPC3. Moreover, HEPC3.1 and HEPC3.4 have ten residues-long CDRH2 loops, which matches the AR3C HCDR2 loop length, but not HEPC3’s, which is nine amino acids long. It appears that in some cases the interactions between HCDR2 and HCDR3 loops may contribute to the stabilization of the bent conformation of the HCDR3 loop (Fig. 6c, clusters 4, 5, and 6). Some other bent HCDR3 loop conformers clearly do not interact with the HCDR2 loop (Fig. 6c, clusters 3 and 8). Overall, the bent conformations of HEPC3.1 and HEPC3.4 appear to often interact with the HCDR2 loop, while the bent conformation of HEPC3 largely does not make these contacts. This observation suggests that HCDR2 loop indeed may contribute to the HEPC3 loop conformation preference.

## Discussion

In this study, we discovered new HCV E2 glycoprotein-binding Abs derived from the *V*_*H*_*1-69* gene and containing a disulfide bond in the HCDR3 loop in the Ab repertoire of individuals with a history of spontaneous clearance of the HCV infection. Conventional sibling search yielded two HEPC3-like Abs, each containing three mutations in the HCDR3 loop. With the help of a P3SM approach, we identified two additional, independently evolved in a different individual, HEPC3-like Abs with low sequence homology. While the sibling Abs demonstrated slightly higher neutralization potency than the ones found with the help of Rosetta, the described structure-based sequence-agnostic discovery of functional Abs in a different donor is important for the Ab research field in general. The P3SM approach broadens the Ab candidate pools that can be surveyed and the resultant increased diversity of the discovered Abs should have a larger impact on our understanding of structural and biophysical determinants of antigen recognition by Abs. However, the full benefit of the P3SM approach is most likely to be achieved when applied to Abs in which interaction with the antigen is dominated by the HCDR3 loop. There is also a significant limitation implied by the current requirement of the exact match of the HCDR3 loop lengths. It might be overcome in the future, for example, with the help of length-independent CDR classes^33^.

The unexpected conformation of the HCDR3 loops of two discovered Abs prompted us to perform a systematic analysis of known structures of Abs with disulfide bonds in the HCDR3 loops. Our analysis suggests that the co-appearance of two amino acids, proline and glycine, in the beginning and toward the end of the loop may increase the propensity for bending in these types of conformationally restricted long HCDR3 loops. Results from our MD simulation and biochemical analyses also lead us to speculate that variability in binding and neutralization efficiency may be related to the flexibility of different HCDR3 loops. We anticipate that continued structural and dynamical insight into long HCDR3 loops will aid rationale epitope-based Ab design.

Despite similarities, there are notable differences between members of this class of *V*_*H*_*1-69* gene-encoded and HCDR3 loop disulfide bond-containing Abs. For example, it has been shown that out of five tested Abs (HEPC3, HEPC74, AR3A, AR3C, and AR3X), only HEPC74 retains almost its full functionality when all somatic mutations are reverted, while both breadth and affinity of other Abs drop dramatically^21,22,25^. Whether somatic mutations in non-HCDR3 regions adjust the recognition of the E2 glycoprotein by fine-tuning the precise placement of the particular HCDR3 loop tip is yet to be determined. Newly discovered members of this class of Abs will contribute to our understanding of this topic. We anticipate that our results will aid future rational lineage-targeting vaccine design initiatives. In particular, the knowledge that both conformations of the HCDR3 loop are widely common will be important to ensure that the desired epitope is available for the Ab approach from both angles.

## Methods

### Ab sequencing

#### Donor C110 (runs 126 and 127)

For subject C110, approximately 1 × 10^7^ PBMCs were enriched for untouched B cells using the EasySep™ Human Pan-B Cell Enrichment Kit (#19554RF, STEMCELL Technologies) on the RoboSep™-S automated cell separator (STEMCELL Technologies). Approximately 9.5 × 10^5^ highly pure B cells were recovered and purity verified by analytic flow cytometric analysis on the Attune™ NxT Flow Cytometer (Thermo Fisher Scientific) prior to total RNA extraction (RNeasy Mini Kit, Qiagen). Total RNA quality was assessed using Agilent TapeStation 4200 System (Agillent) and an RNA integrity number of 7.5 was observed. Total RNA quantity was assessed using the Qubit™ RNA HS Assay Kit and Qubit™ 3.0 Fluorometer (Thermo Fisher Scientific) and a total yield of approximately 750 ng was determined.

All RT-PCR primers used in this study were designed to target only the human IgH recombined locus. The reverse transcription primer consisted of a partial Illumina sequencing adapter, alternating degenerate and spacer regions^34^ (UMI), and a J-gene specific primer^35^. The forward V-gene specific (FR1-region) multiplex PCR primers consisted of a partial Illumina sequencing adapter and FR1 HCV-mAb-specific primer. Specifically, two separate HCV-mAb-specific FR1 primers were designed based on exact nucleotide sequences observed in mAbs HEPC3 and HEPC74 that had been previously isolated and sequence-characterized^16^. A reverse multiplex step-out PCR primer was used to anneal to the Illumina adapter region present in the reverse transcription primer. Forward and reverse adapter-extension primers were used to amplify the target rearrangement region and simultaneously incorporate complete Illumina adapters and sample indexes in the second PCR library preparation reaction. All primers were synthesized and RNase-free HPLC purified (Integrated DNA Technologies). The details of all primer sequences are shown in Supplementary Table 1.

RT-PCR was conducted based on a multi-step approach that incorporates unique molecular identifiers (UMIs) during first-strand cDNA reverse transcription^34^. Briefly, two replicates—one with 75 ng total RNA and the second with 37.5 ng total RNA—were used as an input for reverse transcription. First, reverse transcription was performed using the PrimeScript™ 1st strand cDNA Synthesis Kit (6110A, Clontech) and reverse transcription primers according to the manufacturer’s recommendations. Following cDNA synthesis, samples were subjected to a 0.8X SPRIselect bead cleanup (B23318, Beckman Coulter). Second, multiplex PCR reaction consisted of all purified first-strand cDNA, 500 nM V-gene specific primers, 500 nM 3′ adapter step-out primer, and 1× KAPA HIFI HotStart Uracil +  ReadyMix (KAPA Biosystems, KK2802) in 50 μL was prepared. Thermocycling was performed as follows: 95 °C for 2 min; 9 cycles of 98 °C for 20 s, 60 °C for 45 s, 72 °C for 60 s; 72 °C for 5 min; and 4 °C indefinitely. PCR reactions were then purified using a 0.8X SPRIselect bead cleanup. Third, we performed library preparation by combining all purified multiplex PCR product, 1 μM 3′ Illumina adapter extension primer (Illumina index specific), 1 μM 5′ Illumina adapter extension primer, and 1× KAPA HIFI HotStart ReadyMix (KAPA Biosystems, KK2602) in a 50 μl reaction. Thermocycling was performed as: 95 °C for 5 min; 18 cycles of 98 °C for 20 s, 65 °C for 15 s, 72 °C for 15 s; 72 °C for 5 min; and 4 °C indefinitely. Following library preparation, PCR reactions were cleaned using a double-sided SPRIselect bead cleanup process with 0.5×–0.8× volumes of SPRI beads. After library preparation, libraries were characterized for quality and quantity using the Agilent TapeStation 4200 System (Agillent), Qubit™ dsDNA HS Assay Kit and Qubit™ 3.0 Fluorometer (Thermo Fisher Scientific), and KAPA Library Quantification Kit for Illumina platforms (KK4828, KAPA Biosystems) on an Applied Biosystems™ 7500 Real-Time PCR System (Thermo Fisher Scientific). Two separate next-generation sequencing runs were performed on the Illumina MiSeq platform with a MiSeq Reagent Kit V3 2 × 300 (600 cycle) (MS-102-3003, Illumina) using an input of 12.5 pM with 10% PhiX.

#### Donor C117 (run 47)

For subject C117 total RNA was directly extracted from approximately 1 × 10^7^ PBMCs for each of the four time points available (Supplementary Table 2) (RNeasy Mini Kit, Qiagen).

All RT-PCR primers used in this study were designed to target only the human IgH recombined locus. We utilized only a single VH1 V-gene specific (FR1-region; VH1-FWD-Primer) and J-gene specific primer (JH-RT-REV-Primer) that have been previously described^35^. Our rationale for limiting primer multiplexing to only the VH1-gene-specific family during RT-PCR target enrichment was to specifically enrich for clonal sequences that may belong to previously described VH1-encoded HEPC3 and HEPC74 mAbs^16^. The details of all primer sequences are shown in Supplementary Table 3.

RT-PCR target enrichment was conducted using the SuperScript™ III One-Step RT-PCR System with Platinum™ Taq High Fidelity DNA Polymerase (Invitrogen). Briefly, 2 µL total RNA resulting from each timepoint sample was used as input in separate one-step RT-PCR reactions following the manufacturer’s recommendations. Reactions consisted of 25 µl 2X Reaction Mix (containing 0.4 mM of each dNTP, 2.4 mM MgSO4), 200 nM VH-1 gene-specific primer, 200 nM J-gene specific primer, and 1 µL SuperScript® III RT/Platinum® Taq High Fidelity Enzyme Mix in a 50 µL reaction. Thermocycling was performed as follows: 52 °C for 30 min; 94 °C for 2 min; 40 cycles of 94 °C for 15 s, 56 °C for 30 s, 68 °C for 60 s; 68 °C for 5 min; and 4 °C indefinitely. RT-PCR reactions containing a ~300 bp target fragment were then gel purified using QIAquick Gel Extraction Kit (Qiagen). Library preparation was conducted using the NEBNext® Ultra™ DNA Library Prep Kit following the manufacturer’s recommendations to incorporate Illumina adapter and unique sample index sequences (New England BioLabs). After library preparation, libraries were characterized for quality and quantity using the Agilent 2100 Bioanalyzer System (Agilent), Qubit™ dsDNA HS Assay Kit and Qubit™ 2.0 Fluorometer (Thermo Fisher Scientific), and KAPA Library Quantification Kit for Illumina platforms (KK4828, KAPA Biosystems) on an Applied Biosystems™ 7500 Real-Time PCR System (Thermo Fisher Scientific). A single next-generation sequencing run was performed on the Illumina MiSeq platform with a MiSeq Reagent Kit V3 2 ×300 (600 cycle) (MS-102-3003, Illumina) using an input of 12.5 pM with 15% PhiX.

### Bioinformatics and processing of next-generation sequencing (NGS)

The bioinformatics processing of all NGS data was done using our in-house bioinformatics sequence processing pipeline. An outline of the processing steps is provided below.

#### Quality inspection and generation of full length reads

Paired end (PE) reads generated using the Illumina sequencing platform were assessed using the FASTQC^36^ toolkit (version 0.11.5) to make a determination of the quality of the run. For those sequencing runs where the average Phred score was 20 or greater by inspection of the FASTQC base sequence quality plot, we proceeded to remove primers from the framework regions.

#### Removal of framework primers

BIOMED-2 framework primers from the PE reads were removed using the program FLEXBAR v3.0^37^. The minimum overlap for each primer (*–adapter-min-overlap*) was set to 14 and the error rate allowed for each primer (*–adapter-error-rate*) was set to 0.3.

#### Merging PE reads to generate full-length contigs

After the removal of framework primers, the PE reads were merged using the program USEARCH v9.1^38^. The overlap region (*-fastq_minovlen*) was set to 15 nucleotides and the maximum number of differences in the overlap region (*-fastq_maxdiffpct*) was set to 10. Following merging of PE reads, we analyzed the FASTQC plots to determine the quality of the merged reads.

#### Germline gene assignment and definition of CDR3 regions

We used PyIR^39^ to process the contigs generated with USEARCHv9.1. PyIR parses IgBLAST^40^ output to obtain germline gene assignments, the nucleotide sequence for the variable region (V(D)J segment), somatic mutation counts, and the CDR3 region.

#### Filtering reads using MongoDB

Processed reads were imported into our local MongoDB cluster for quality control filtering. Each read was subjected to a series of knowledge-based sieves in the following order: (1) Removal of any read that had an average Phred score of <30; (2) Removal of any read that had an E-value larger than 10^−6^ for IGHV/IGHJ germline assignments; (3) Removal of any read that did not have a defined CDR3; (4) Removal of any read containing a stop codon; (5) Removal of any read with more than four contiguous amino acid insertions in the framework region; (6) Removal of any read that was out of frame at the junction region; and (7) Removal of reads for which the nucleotide length from framework 1 through framework 3 was <250 nucleotides. All remaining reads were considered high-quality and labeled as productive reads. We did not remove reads with undefined bases unless they occurred in the CDR3 region.

#### De-replication of productive reads using MongoDB

To remove redundancy in the data, multiple copies of the same V(D)J segment were removed from the productive pool of sequences. The number of times a V(D)J segment appeared in the data set was retained for downstream rarefaction analysis. This process of de-replication effectively reduced the size of the data set that needed to be integrated into the database.

The results of sequencing data processing are summarized in Supplementary Table 4.

### In silico protein modeling

Before the start of the computational analysis, the crystal structures of the bNAbs HEPC3 and AR3C in complex with HCV envelope glycoprotein E2 (PDB IDs: 6MEI and 4MWF) were relaxed using Rosetta Relax application^41^ using the following flags:


-flip_HNQ-no_optH false-relax:constrain_relax_to_start_coords-relax:ramp_constraints false-nstruct 50-ex1-ex2-use_input_sc


The structure with the lowest total score out of ten generated structures was used for further work.

To generate a Position-Specific Structure Scoring Matrix (P3SM) for HEPC3, 1200 sequences of 19-residue HCDR3 loops with cysteine amino acid residues at positions 8 and 13 were selected randomly from a database of NGS data containing Ab sequences from PBMCs of healthy individuals. Each of these 1200 sequences was threaded using Rosetta onto the HEPC3 structure in the context of E2 and then relaxed to generate ten models for each HCDR3 sequence using the following XML script:

<ROSETTASCRIPTS>

    <SCOREFXNS>

        <ScoreFunction Name="tal14" weights="talaris2014"/>

        <ScoreFunction Name="tal14_cart" weights=" talaris2014_cart"/>

    </SCOREFXNS>

    <FILTERS>

    </FILTERS>

    <TASKOPERATIONS>

        <ReadResfile Name="rr" filename="%%resfile%%"/>

        <InitializeFromCommandline Name="ifcl"/>

        <RestrictToRepacking Name="rtr"/>

    </TASKOPERATIONS>

    <MOVERS>

        <PackRotamersMover Name="pr" scorefxn=" tal14" task_operations="ifcl,rr"/>

        <MinMover Name="min" scorefxn="tal14" chi="1" bb="1" jump="1" tolerance="0.01"/>

        <FastRelax Name="relax" scorefxn="tal14_cart" cartesian="1" repeats="1" task_operations="ifcl,rtr"/>

        <InterfaceAnalyzerMover Name="iface_analyzer" scorefxn=" tal14_cart" packstat="0" pack_input="0" pack_separated="1" fixedchains="H,L" tracer="0" interface_sc="1"/>

    </MOVERS>

    <PROTOCOLS>

            <Add mover_name="pr"/>

            <Add mover_name="relax"/>

            <Add mover="iface_analyzer" />

    </PROTOCOLS>

    <OUTPUT scorefxn="tal14_cart"/>

</ROSETTASCRIPTS>

For each of the 1200 threaded sequences, the five models with the lowest total Rosetta energy were selected for further analysis (a total of 6000 models). One-hot encoded loop sequence data were combined with either loop total score or protein complex interface score to estimate a score for each of the 20 amino acids at every position in the HCDR3 using Ridge regression model as implemented in *sklearn.linear_model.Ridge*. If none of the analyzed sequences contained a particular amino acid in a particular position of the loop, a zero value was assigned to that position. The same procedure was performed for AR3C using a set of 1200 20-residue HCDR3 loop sequences with cysteine amino acid residues at positions 9 and 14.

All preselected 19- and 20-long HCDR3 sequences from two individuals with a history of spontaneous clearance of the HCV infection were subsequently scored by appropriate P3SMs. Scoring by P3SM means summing the score values for all amino acids in the loop. The top-scoring sequences (all unique sequences among 20 or 10 (~15%) top sequences ranked by each P3SM (total score-based and interface score-based) for HEPC3-like or AR3C-like sequences, correspondingly, Supplementary Tables 5 and 6) were threaded onto the corresponding HEPC3 or AR3C complex structure. The total score of the complex, predicted binding affinity, HCDR3 root-mean-square deviation (RMSD) to a template Ab, shape complementarity, and the number of unsatisfied hydrogen bond partners were calculated for each of the sequences and compared to the corresponding values of the template Ab. We used these values to guide the selection of sequences for testing aiming for similar or better scores compared to the template Ab but due to the small number of the analyzed sequences, no strict selection cutoffs were implemented. We also visualized and checked all resulting models manually in PyMOL.

### Molecular dynamics (MD) simulations

MD simulations were performed for six Abs: HEPC3, HEPC3-PG (HEPC3-V_97_P-N_100G_G), HEPC3.1, HEPC3.1-VN (HEPC3.1-P_97_V-G_100G_N), HEPC3.4, and HEPC3.4-VN (HEPC3.4-P_97_V-G_100G_N). Each of these Abs was simulated from two starting conformations: (1) the HEPC3 crystallographic pose with the straight HCDR3 loop; (2) the HCDR3.1/3.4 crystallographic pose with the bent HCDR3 loop. The HEPC3 bent HDCR3 loop conformation was modeled with multi-template Rosetta comparative modeling (RosettaCM) using both HEPC3.1 and HEPC3.4 structures as templates. Similarly, the HEPC3.1 and HEPC3.4 straight HCDR3 loop conformation models were generated with RosettaCM using HEPC3 as a template. The crystallographic structures of HEPC3 straight and HEPC3.1 and HEPC3.4 bent conformations were relaxed in Rosetta prior to MD simulation. The best scoring poses of the Rosetta relaxed crystal structures or RosettaCM models were used to initiate all MD simulations.

Systems were parameterized with Leap in AmberTools20^42^. Simulations were performed with Amber20 using the Particle Mesh Ewald MD CUDA (PMEMD.cuda) application^42^ and ff19SB force field^43^. Structures were solvated in a rectangular box of OPC explicit solvent neutralized with Joung–Cheatham monovalent ions^44,45^. Abs were buffered on all sides with 12.0 Å solvent. Hydrogen mass repartitioning was performed on solute atoms to allow a simulation timestep of 4.0 fs^46^.

Minimization proceeded in three stages: (a) The system was minimized with 5000 cycles of steepest descent followed by 10,000 steps of conjugate gradient descent (CGD) while protein atoms were restrained with a force constant of 5.0 kcal mol^−1^ Å^−2^; (b) the system then underwent 2000 cycles of steepest descent followed by 8000 steps CGD minimization while buffer atoms were restrained with a force constant of 5.0 kcal mol^−1^ Å^−2^; (c) all restraints were removed from the system for 500 steps steepest descent followed by 9500 steps of CGD minimization.

Following minimization, covalent bonds to hydrogen atoms were constrained with the SHAKE^47^ algorithm implemented in Amber20 with a tolerance of 0.0000001. Periodic boundary conditions were imposed on the system and the Particle Mesh Ewald (PME) approximation was employed for long-range interactions beyond 9.0 Å. The temperature was controlled using Langevin dynamics with a collision frequency of 5 ps^−1^ during heating and 2 ps^−1^ in production simulations. A unique random seed was used for each Langevin dynamics simulation. Systems were slowly heated to 100 K in the canonical (NVT; constant number of particles, temperature, and volume) ensemble over 25 ps with a 0.1 fs timestep. Subsequently, systems were heated in the NPT (isothermal-isobaric) ensemble at 1.0 bar with isotropic position scaling from 100 to 300 K over 250 ps and 1 fs timestep. Pressures were maintained with a Monte Carlo barostat. Production simulations were run in the NPT ensemble at 1.0 bar and 300 K with an integration timestep of 4.0 fs. A total of six independent trajectories of 12 systems (two conformations each for HEPC3, HEPC3-PG, HEPC3.1, HEPC3.1-VN, HEPC3.4, and HEPC3.4-VN) were run for 5.0 µs. The total production simulation time was therefore 360.0 µs. Simulation trajectory frames containing solute atoms were collected every 10 ps for a total of 500,000 frames per trajectory.

### MD simulation trajectory analysis

To compare the configurational sampling of Ab HCDR3 loops, every 10th simulation frame for all systems were converted into trajectories containing only the common backbone atoms between all proteins. These common atom trajectories were converted into feature trajectories consisting of HCDR3 loop residue pair closest heavy atom distances, angles, and dihedral angles (see Supplementary Data 1 for lists of all 1497 features). The resulting feature space for each frame in all feature trajectories was transformed with time-lagged independent component analysis (TICA). TICA was performed with PyEMMA2^48^ using a lag time of 10 steps (1 ns) using all the independent feature trajectories (3,600,000 frames). TICA dimensions were calculated to account for 95% of the cumulative kinetic variance. The resulting time-lagged independent components (ICs) represent the common kinetic variance across all simulations.

Subsequently, the feature trajectories of each independent simulation were mapped to the previously computed TICA dimensions and aggregated within each of the 12 groups corresponding to the MD simulation systems (300,000 frames per group). All groups were plotted as 2D-histograms along the first two ICs of the common TICA space. Each histogram dimension was discretized with 200 bins. The color scale used to represent the bin density is log-scaled and normalized with respect to the highest density bin across all trajectories. For reference, the crystallographic structures of HEPC3, HEPC3.1, and HEPC3.4 were also projected along the first two ICs of the common TICA space.

### Antibody genes cloning, expression, and purification

Genes encoding the V_H_ domains of chimeric antibodies that recapitulate the computationally tested proteins (designated HEPC3.1_HCDR3_ – HEPC3.8_HCDR3_, AR3C.1_HCDR3_ – AR3C.2_HCDR3_) were synthesized as gBlocks gene fragments (IDT) and cloned into the pTT5-based vectors (NRC Biotechnology Research Institute). The same platform was used to clone native V_H_ domains. HEPC3-derived constructs (containing 19-residue HCDR3 loops) and HEPC3 sibling heavy chain sequences were paired with the HEPC3 V_L_ and AR3C-derived constructs (containing 20-residue HCDR3 loops) were paired with AR3C V_L_. IgGs or His-tagged Fabs for structural studies were produced in Expi293F cells by co-transfecting with appropriate heavy- and light-chain plasmids. HiTrap Protein A HP or HisTrap FF columns (GE Healthcare) were used to isolate IgGs or His-tagged Fabs from filtered culture supernatants followed by purification by size exclusion chromatography (SEC).

### Expression and purification of E2 glycoproteins

His-tagged HCV E2 glycoproteins (residues 384–643) for ELISA binding assays were expressed by transiently transfecting Expi293F (American Type Culture Collection) or HEK293-6E (National Research Council of Canada) cells and purified from clarified supernatants using a HisTrap FF column (GE Healthcare) followed by SEC on a Superdex 200 Increase 10/300 GL column (GE Healthcare) to separate monomeric E2 proteins from oligomeric species.

### ELISA binding analysis

Soluble forms of E2 ectodomains (residues 384–643) were coated overnight onto 96-well plates (Corning) at 1 μg/mL. 1a157 E2ecto ΔFRLY contains mutations T425A, L427A, N428A, S432A, G436A, W437A, G530A, D535A that disable binding of HEPC3-like Abs that bind to the AR3 antigenic site. The specificity and selectivity of E2ecto ΔFRLY knockout variant were verified in ELISA binding assay (Fig. 2c, d). After blocking the plates with 1% goat serum and 1% powdered milk in TBST buffer (TBS with 0.05% Tween-20) for 1 h, purified IgGs were assayed in duplicate at fourfold serial dilutions, starting at 10 µg/mL. For detection, goat anti-human IgG horseradish peroxidase-conjugated secondary antibody (Southern Biotech, 1:4000 dilution) and 1-Step Ultra TMB-ELISA substrate (Thermo Fisher Scientific) were used. The signal was measured by reading the optical density at 450 nm after stopping the reaction with 1 M HCl. A non-linear regression analysis was performed on the resulting curves using Prism version 5 (GraphPad) to calculate EC_50_ values and area under the curve (AUC).

### HCVpp neutralization assays

HCV pseudoparticles (HCVpp) were produced by lipofectamine-mediated transfection of HCV E1E2 and pNL4-3.Luc.R-E-plasmids into HEK293T cells as described^49,50^. A panel of 19 heterologous genotype 1 HCVpp and genotype 2–6 HCVpps have been described previously^5,51,52^. Neutralization assays were performed as described^53^. Briefly, IgGs were serially diluted five-fold, starting at a concentration at 100 µg/ml, and incubated with HCVpp for one hour prior to addition to Hep3B hepatoma cells (American Type Culture Collection). Luciferase activity was measured after three days and compared to that of HCVpp in media alone to calculate percent neutralization. A non-linear regression analysis was performed on the resulting curves using Prism version 5 (GraphPad) to calculate IC_50_ values.

### Crystallization, data collection, and structure determination

Commercially available crystallization screens (Hampton Research and Molecular Dimensions) were used to screen initial crystallization conditions by vapor diffusion in sitting drops. HEPC3.1 crystals were grown using 0.2 µL of protein complex in TBS and 0.2 µL of mother liquor (2.0 M ammonium sulfate) and cryoprotected in mother liquor supplemented with 20% (w/v) glycerol. HEPC3.4 crystals were grown using 0.2 µL of protein complex in TBS and 0.2 µL of mother liquor (3.0 M sodium formate, sodium acetate trihydrate pH 4.6) and cryoprotected in mother liquor supplemented with 15% (w/v) glycerol. X-ray diffraction data from cryopreserved crystals were collected at the Stanford Synchrotron Radiation Lightsource on beamline 12–2 using a PILATUS 6 M detector. Images were processed and scaled using iMosflm^54^ and Aimless as implemented in the CCP4 software suite^55^. The HEPC3.1 and HEPC3.4 structures were solved by molecular replacement using the HEPC3 (PDB 6MED) and HEPC3.1 structures as search models. The models were refined and validated using Phenix.refine^56^. Iterative manual model building and corrections were performed using Coot^57^. The quality of the final models was examined using MolProbity^58^. The final statistics of the structures are shown in Supplementary Table 7. Models were superimposed and figures were rendered using the PyMOL (Schrodinger, LLC).

### Human research participants

Previously banked peripheral blood mononuclear cells from two hepatitis C virus-infected individuals were provided as de-identified, identity-unlinked samples from the Baltimore Before and After Acute Study of Hepatitis (BBAASH) cohort at the Johns Hopkins University School of Medicine. Participants sign written informed consent to participate in the cohort. This study was approved by the Institutional Review Board of the Johns Hopkins University School of Medicine.

### Reporting summary

Further information on research design is available in the Nature Research Reporting Summary linked to this article.

## Supplementary information

Supplementary Information

Description of Additional Supplementary Files

Supplementary Data 1

Reporting Summary

## Data Availability

The crystal structures reported in this paper has been deposited to the Protein Data Bank under accession numbers 7U0B and 7U0C. Other structures used in this study are available in the Protein Data Bank under accession numbers 2FB4 (Kol)^59^, 6C6Z (CDC2-C2)^60^, 5BV7 (27C3)^61^, 4NZU (13PL)^62^, 3EYF (8F9)^63^, 6IEA (R13)^64^, 6MED (HEPC3)^25^, 5V7U (dmCBTAU)^65^, 6VBQ (DH822)^66^, 6BKB (AR3A)^21^, 6MEF (AR3C)^25^, 6Q19 (652Int)^67^, 6WO4 (HC11)^23^, 6URH (AR3X)^22^, 4PTU (C05)^68^, 6APC (ADI)^69^, 5IG7 (1002-1E01)^70^, 6MEE (HEPC74)^70^, 6BLI (CB002.5)^71^, 6Q1J (H2227)^67^, 6PBV (Fab668)^72^, 6UOE (3–25)^73^, 6Q1G (H1244)^67^, 6Q0E (652UCA)^67^, 4XAK (m336)^32^. NGS data for Ab libraries were deposited to the Sequence Read Archive under accession number PRJNA813433. Rosetta Ab models as well as coordinate and restart files for all MD trajectories can be accessed from GitHub at https://github.com/meilerlab/HEPC3. Source data are provided with this paper.

## Source data

Source Data
