# Supplementary material for: Computational identification of HCV neutralizing antibodies with a common HCDR3 disulfide bond motif in the antibody repertoires of infected individuals
Source: Nat Commun. 2022 Jun 8;13:3178. doi: 10.1038/s41467-022-30865-9 (PMC9177688; doi:10.1038/s41467-022-30865-9)
Supplement: Supplementary file 1 — Supplementary Information [file 41467_2022_30865_MOESM1_ESM.pdf]

Supplementary information:
Computational identification of HCV neutralizing antibodies with a common HCDR3 disulfide bond motif in the antibody repertoires of infected individuals

N. Bozhanova *et al.*

	HEPC3	HEPC74	AR3A	AR3C	AR3X
**HC11**	32%<br>ARDGVRYCGGGRCYNWFDP<br>AMEVPGFCRGGSCSGYMDV	35%<br>ARDLLKYCGGGNCHSLLVDP<br>AMEVPGFCRGGSC-SGYMDV	40%<br>VRPETPRYCSGGFCYGEFDN<br>-AMEVPGFCRGGSCSGYMDV	40%<br>VRSVTPRYCGGGFCYGEFDY<br>AMEV-PGFCRGGSCSGYMDV	52%<br>ARENKFRYCRGGSCYSGAFDM<br>AME-VPGFCRGGSC-SGYMDV
**AR3X**	52%<br>AR-DGVRYCGGGRCYNW-FDP<br>ARENKFRYCRGGSCYSGAFDM	43%<br>AR-DLLKYCGGGNCHSLLVDP<br>ARENKFRYCRGGSCYSGAFDM	52%<br>VRPETPRYCSGGFCY-GEFDN<br>ARENKFRYCRGGSCYSGAFDM	52%<br>VRSVTPRYCGGGFCY-GEFDY<br>ARENKFRYCRGGSCYSGAFDM	
**AR3C**	55%<br>AR-DGVRYCGGGRCYNWFDP<br>VRSVTPRYCGGGFCYGEFDY	40%<br>ARDLL-KYCGGGNCHSLLVDP<br>VRSVTPRYCGGGFCYGEF-DY	80%<br>VRPETPRYCSGGFCYGEFDN<br>VRSVTPRYCGGGFCYGEFDY		
**AR3A**	50%<br>AR-DGVRYCGGGRCYNWFDP<br>VRPETPRYCSGGFCYGEFDN	35%<br>ARDLL-KYCGGGNCHSLLVDP<br>VRPETPRYCSGGFCYGEF-DN			
**HEPC74**	55%<br>ARDGVRYCGGGRCYNWF-DP<br>ARDLLKYCGGGNCHSLLVDP				

Supplementary Figure 1. The pairwise similarity of HCDR3 loop sequences between six chosen E2 front layer-specific bNAbs. Each cell shows an alignment of the amino acid sequences of the two corresponding HCDR3 loops (IMGT definition). Identical amino acids are colored blue. The percentage of identity is calculated as a ratio of the number of identical amino acids between the two sequences and the length of the longest loop in the pair. The only pair of Abs that fulfills the traditionally used 75-80% identity cutoff for HCDR3 loops in sibling search is highlighted with green.

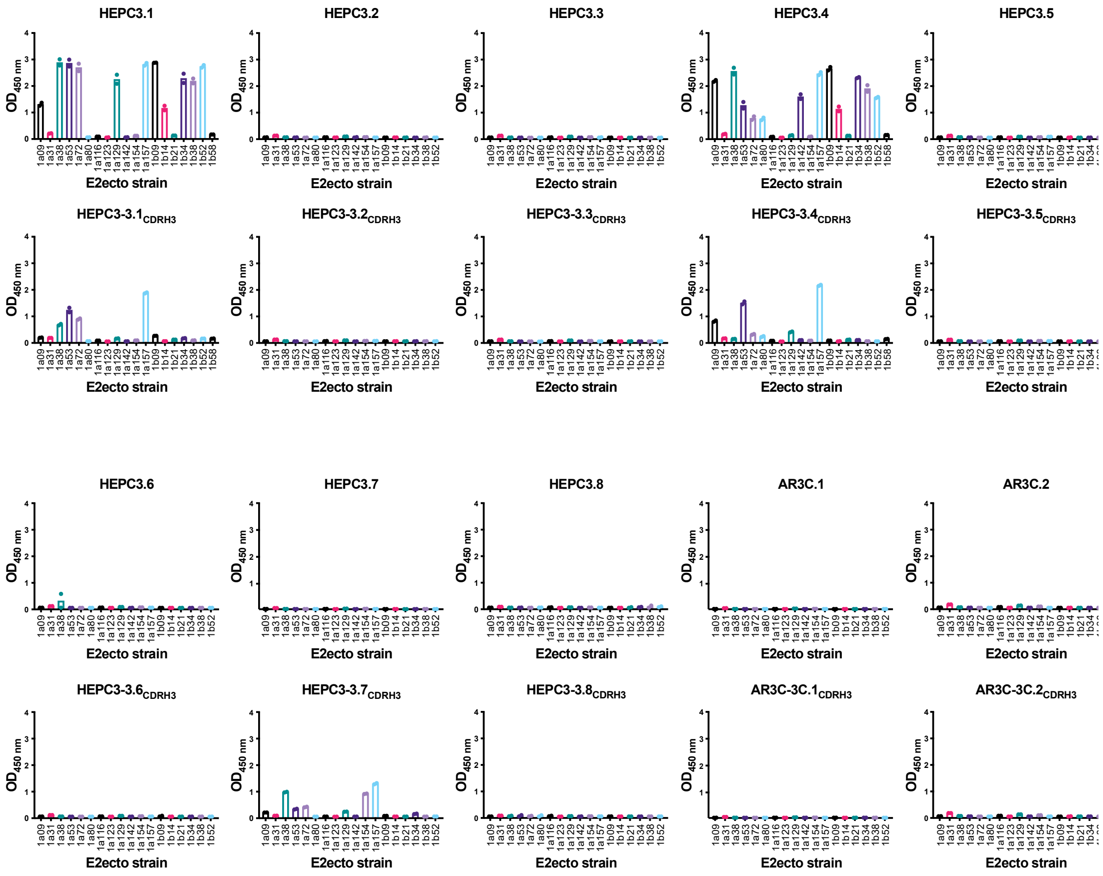

Supplementary Figure 2. Binding of HEPC3- and AR3C-like Abs to a panel of genotype 1 E2 glycoproteins. Data are presented as means of technical duplicates, and individual data points are indicated. Source data are provided in the Source Data file.

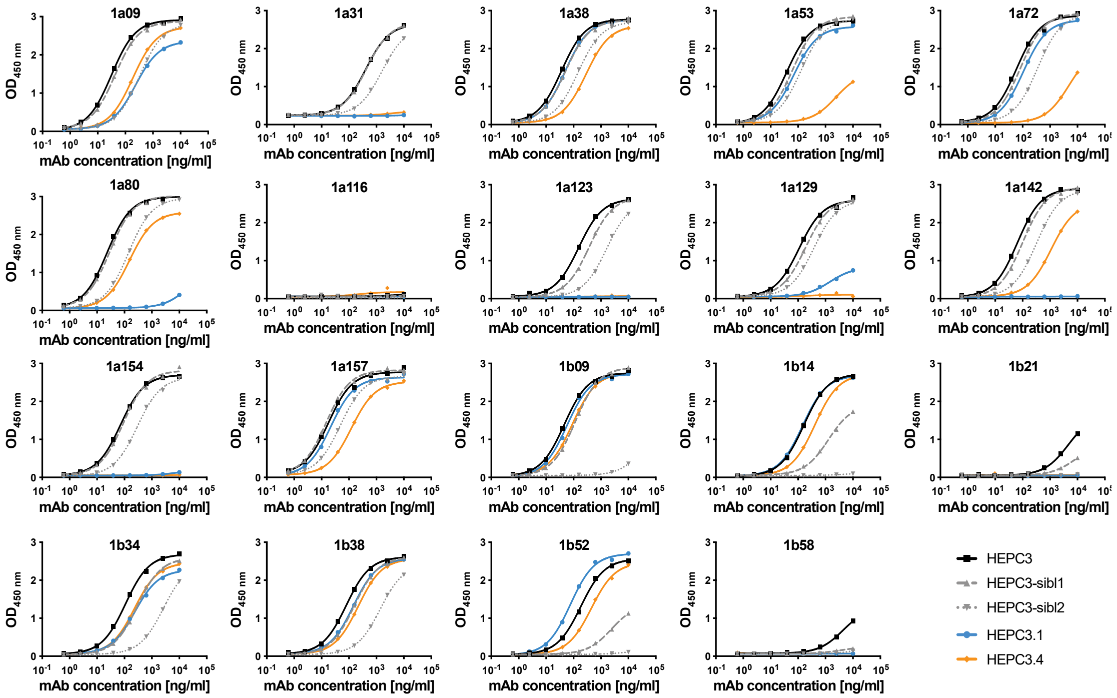

Supplementary Figure 3. Binding of HEPC3.1, HEPC3.4, HEPC3-sibl1, and HEPC3-sibl2 Abs to a panel of 19 genotype 1 E2 glycoproteins. Values shown are means of technical duplicates. One experiment representative of two independent experiments is shown. Source data are provided in the Source Data file.

```
                                      HCDR1                  HCDR2                                                    HCDR3
     HEPC3 QVQLVQSGAE VKKPGSSVKV SCKASGGTLN SYEITWVRQA PGQGLEWMGG ITPIFET-TY AQKFQGRVTI TADESTSTTY MELSSLRPED TAVYYCARDG V-RYCGGGRC Y--NWFDPWG QGTLVTVSS
  Rand-VH_1 .......... .......... ........FS ..A.S..... .......... .I...G.AN. .......... ........A. .......S.. ........EI P-G-.S..S. .SAA...... .........
  Rand-VH_2 .......... .......... ........FS ..A.S..... .......... .I...G.AN. .......... ........A. .......S.. .........H EWG..S..S. --N....... .........
  Rand-VH_3 .......... .......... ........FS ..A.S..... .......... .I...G.AN. .......... ........A. .......S.. ........GT L--..S..S. .ST....... .........
  Rand-VH_4 .......... .......... ........FS ..T.S..... .........R .I..LGIAN. .......... ...K....A. .......S.. ........YR L-G..S..S. .HN....... .........
  Rand-VH_5 .......... .......... ........FS ..A.S..... .......... .I...G.AN. .......... ........A. .......S.. ........AL L--..S..S. .H-....... .........
  Rand-VH_6 .......... .......... ........FS T.A.S..... .......... .I.V.R.TN. .......... ........AF ..MN...S.. .........S L-E..S..G. .--Q...L.. P........
  Rand-VH_7 .......... ....WA.... ........FS ..A.S..... .......... .I...G.AN. .......... ........A. .......S.. .........L G-GL.S..S. -SGWGM.V.. ...T.....
  Rand-VH_8 .......... ....EA.... ......Y.FT ..A.S..... .......... .I...G.AN. .......... ........A. .......S.. ........QN S--..S..S. .S-D...... .........
  Rand-VH_9 .......... .....A.... ........FS ..A.S..... .......... .I...G.AN. .......... .......... ....R..SD. .....G..VP R-...S..S. H--SG..Y.. .........
 Rand-VH_10 .......... ....WA.... ........FS ..A.S..... .......... .I...G.AN. .......... ........A. .......S.. ........GS P--..S..S. .SA.-..D.. .........
```

Supplementary Figure 4. Alignment of the amino acid sequences of the heavy chain variable regions of bNAb HEPC3 with ten Abs containing C-X-G-G-X-C HCDR3 motif randomly selected from the Ab repertoire of patients who spontaneously cleared HCV infection.

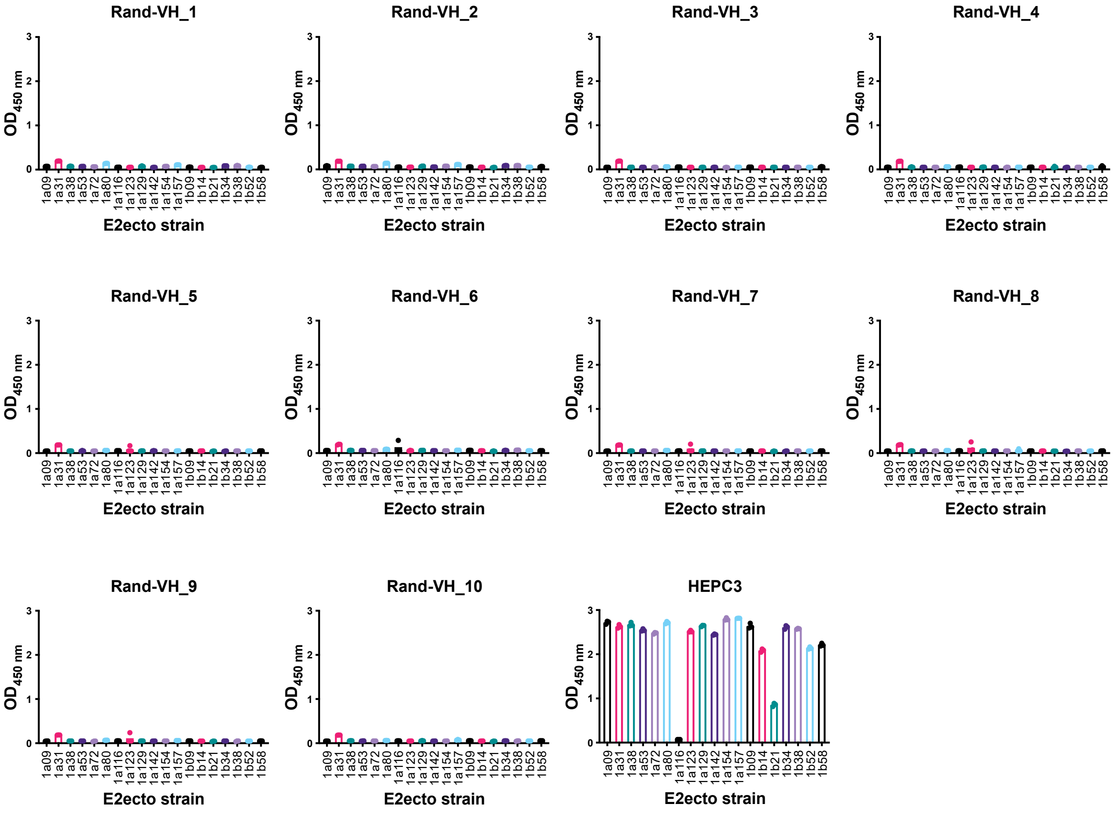

Supplementary Figure 5. Binding of Abs containing C-X-G-G-X-C HCDR3 motif randomly selected from the Ab repertoire of patients who spontaneously cleared HCV infection to a panel of genotype 1 E2 glycoproteins. Data are presented as means of technical triplicates, and individual data points are indicated. Source data are provided in the Source Data file.

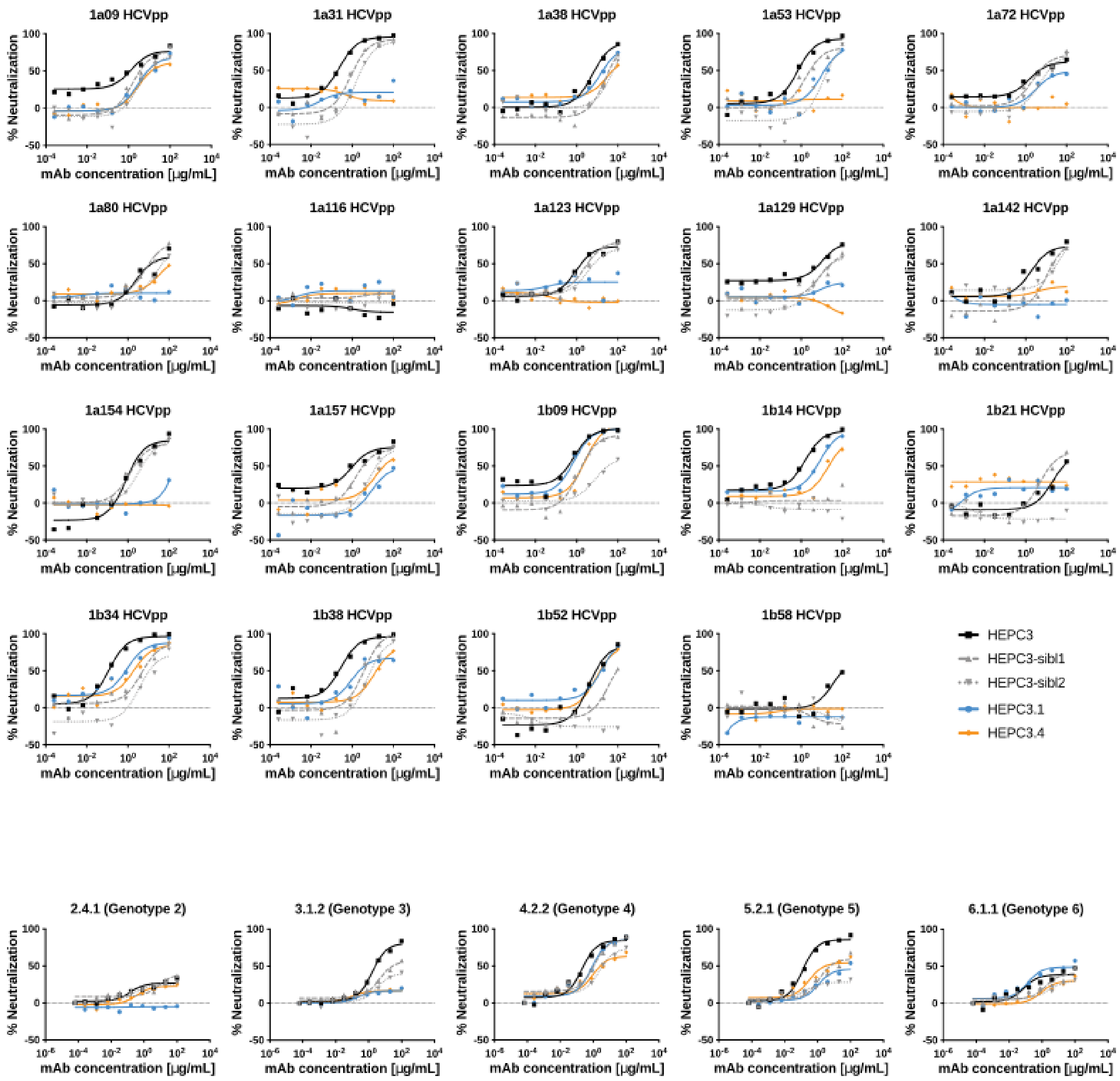

Supplementary Figure 6. Neutralization activities of HEPC3.1, HEPC3.4, HEPC3-sibl1, and HEPC3-sibl2 Abs against a panel of genotype 1-6 HCVpp. Values shown are means of technical duplicates. Source data are provided in the Source Data file.

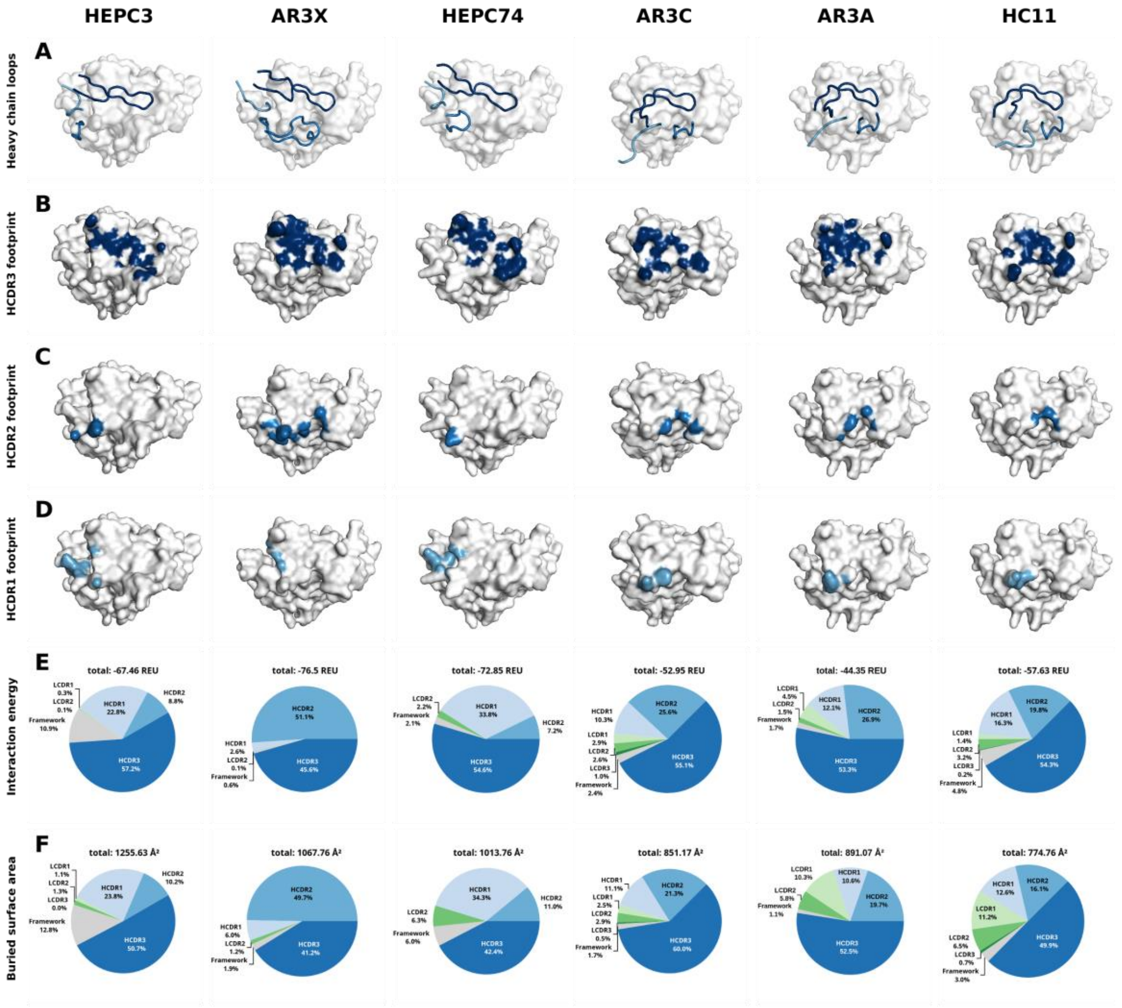

Supplementary Figure 7. Summary of the Ab-antigen interactions of the straight (HEPC3, AR3X, HEPC74) and bent (AR3C, AR3A, HC11) HEPC bNAbs. (**A**). Positions of the HCDR1, HCDR2, and HCDR3 loops of the Abs in the Ab-antigen complexes. The glycoprotein E2 is shown as a surface. The HCDR1, HCDR2, and HCDR3 loops are shown as light blue, blue, and dark blue cartoons, respectively. (**B-D**) Footprints of the HCDR1 (**D**), HCDR2 (**C**), and HCDR3 (**B**) loops. The glycoprotein E2 is shown as a surface. The antigen atoms located within 4 Å from any corresponding HCDR atom are colored. (**E-F**) Pie charts demonstrating relative contribution of different Ab parts (HCDR1, HCDR2, HCDR3, LCDR1, LCDR2, LCDR3, and framework) to antigen binding. (**E**) Decomposition of the Ab-antigen interaction energy calculated in Rosetta. (**F**) Relative contribution of different Ab parts to buried surface area. PDB IDs of the used structures: HEPC3: 6MEI, AR3X: 6URH, HEPC74: 6MEH, AR3C: 4MWE, AR3A: 6BKB, HC11: 6WO4.

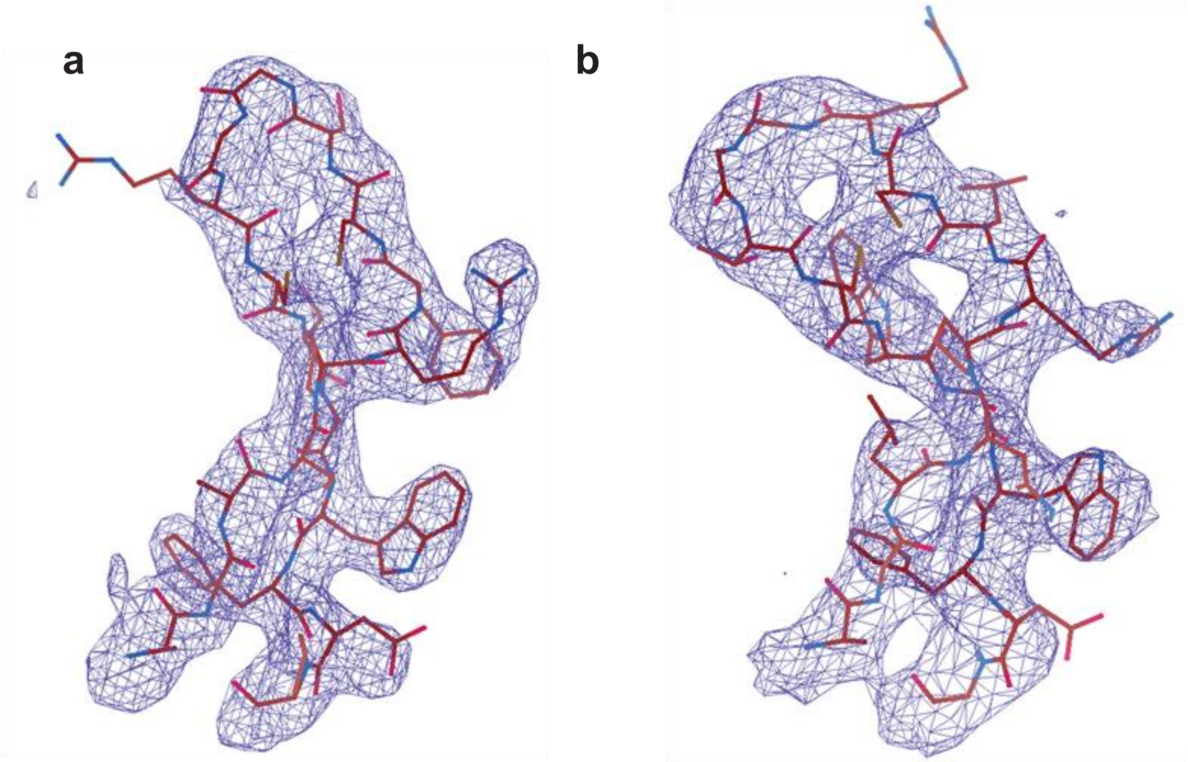

Supplementary Figure 8. Close-up views of CDRH3 density maps for HEPC3.1 (a) and HEPC3.4 Fabs (b). Blue mesh represents the 2Fo-Fc density map generated in Coot by masking 2Fo-Fc map outside the CDRH3 region. The resulting masked maps were contoured at 0.37 e/Å^3 (HEPC3.1) and 0.42 e/Å^3 (HEPC3.4).

Supplementary Table 1. Primer sequence information

Step	Primer Name	Primer Sequence
Reverse transcription	Human IgH RT primer with UMI	TTGGCACCCGAGAATTCCACTGHHHHHACAHHHHHACAHHHHNCTTACCTGAGGAGACGGTGACC
Multiplex PCR	5′ HEPC74-FR1	CGTTCAGAGTTCTACAGTCCGACGATCGTCCTCGGTAAAGGTCTCCTGCACG
	5′ HEPC3-FR1	CGTTCAGAGTTCTACAGTCCGACGATCGTCCTCGGTGAAGGTCTCCTGCAAG
	3′ Adapter step-out primer	ACTGGAGTTCCTTGGCACCCGAGAATTCCACTG
Library preparation	5′ Illumina Adapter Extension Primer	AATGATACGGCGACCACCGAGATCTACACGTTCAGAGTTCTACAGTCCGACGATC
	3′ Illumina Adapter Extension Primer w/ Sample Index	CAAGCAGAAGACGGCATACGAGATXXXXXXGTGACTGGAGTTCCTTGGCACCCG

Supplementary Table 2. Donor *C117* timepoint information

Timepoint ID	Timepoint Date	# PBMCs Used
U1	11/18/2004	1×10^7
U2	2/10/2005	1×10^7
U3	5/12/2005	1×10^7
U4	11/2/2005	1×10^7

Supplementary Table 3. Primer sequence information

Step	Primer Name	Primer Sequence
One-step RT-PCR	VH1-FWD-Primer	GGCCTCAGTGAAGGTCTCCTGCAAG
	JH-RT-REV-Primer	CTTACCTGAGGAGACGGTGACC

Supplementary Table 4. Summary of results of antibody gene repertoire sequencing

Run ID	Total reads	Unique productive reads	Unique clonotypes	Unique HCDR3s
47	8,558,961	2,933,659	825,905	631,114
126	20,665	9,575	9,158	4,106
127	22,326	5,585	5,311	3,709

Supplementary Table 5. Top HEPC3-like sequences and their ranks. The names of the tested sequences are specified in parentheses.

Total score-based P3SM		Interface score-based P3SM	
ARDSLRYCSGGSCYRGMDV (HEPC3.7)	-42.97	ATLHPRLCRGGTCWGWFDP (HEPC3.4)	-52.41
ARDSLGYCSGGSCYRGMDV	-40.56	ARDRVRYCSGGSCYSAFDY	-52.26
ARDSLGYCSGGSCYRGMVV	-39.92	AGALPRFCSGGRCSGWFDP (HEPC3.1)	-51.81
ARDRVRYCSGGSCYSAFDY	-39.92	ASRRRRYCSGGSCPLSDDI	-51.73
ARDRTEDCSGGSCYSALDY	-39.29	**HEPC3**	-50.79
ARSLARYCSGGSCYDGMDV (HEPC3.2)	-39.26	ATIEWGYCSGGSCYRPHDY (HEPC3.8)	-50.53
ARWERDYCSGGSCYYGMDV	-39.04	ARVQQRYCSGGSCPHAFDI	-50.53
ARDGLGYCSGGSCYSGLDY	-38.89	AALMDRYCSGGSCYDAFDI	-50.26
ARYLPRYCSGGSCYNYFDY (HEPC3.5)	-38.23	ARVPRRYCSGGSCHSGFDY	-49.67
AALEALYCSGGSCYSNFDY	-38.22	ARDPVRYCSGGSCPYYFDY (HEPC3.6)	-49.66
ARWERDYCSGGSCYGGMDV	-37.98	AIDRAAYCSGGSCLLGMDV	-49.56
ARVQLGYCSGGSCYYGMDV	-37.9	ARDSLRYCSGGSCYRGMDV (HEPC3.7)	-49.56
ARTYPGYCSGGSCYNGMDV	-37.39	ARYLPRYCSGGSCYNYFDY (HEPC3.5)	-49.37
ATDFLGYCSGGSCYPYSDY	-37.35	ARSLARYCSGGSCYDGMDV (HEPC3.2)	-49.33
**HEPC3**	-37.1	ARDRAAYCSGGSCLLGMDV	-49.32
ARAKTTYCSGGSCYPYFDY	-36.92	ARVQQRSCSGGSCPDAFDI	-49.29
ARDSLGYCSGGSCSYGMDV	-36.91	ARERMGYCSGGSCLTYFDY	-49.14
ARDLPHRCSGGSCYGYFDL	-36.68	ASDRGTYCSGGDCYGGFDL (HEPC3.3)	-49.11
ARWGRDYCSGGSCYGGMDV	-36.66	ARTYPGYCSGGSCYNGMDV	-49.09
ARDEVGYCGGGSCYNWLAP	-36.64	ATGFLGYCSGGSCPQPFDY	-49.03
ATGSLGGCSGGSCYGTMDV	-36.51	ASSPLGYCSGGSCFDAFDI	-48.96

Supplementary Table 6. Top AR3C-like sequences and their ranks. The names of the tested sequences are specified in parentheses.

Total score-based P3SM		Interface score-based P3SM	
ARSQISNYCSGGSCYSASDY	-36.41	ARVPTPRYCSGGSCHNAFDI	-37.56
**AR3C**	-36.1	ARAGYPGYCSGGSCFNWFDP(AR3C.1)	-37.12
AKNQHPGYCSGGSCYSSFDY (AR3C.2)	-35.47	ARVRYINNCSGGSCYYYFDY	-36.93
ARVAVMGYCSGGSCYSGFDY	-34.11	AREGYDYDCSGGSCHNWFDP	-36.54
ARDGDIGYCSGGSCYSGLDY	-34.04	AKNQHPGYCSGGSCYSSFDY	-36.53
ARDASPGYCSGGSCYSTLDY	-33.91	**AR3C**	-36.37
ARDRDTSYCSGGSCYSYFDY	-33.51	ARVRRMRDCGGGSCYSWFDP	-36.23
ARDVSPGYCSGGSCYSTLDY	-33.42	AREGYDYDCSGGSCHNWSDP	-36.12
AREGTGGYCSGGSCYSGMDV	-33.34	ARGPQTRYCSGGSCADAFDI	-36.07
ARDVSPGYCSGGSCYSTLYY	-32.62	AERYFGRYCSGGSCYSPFDY	-35.9

Supplementary Table 7. Data collection and refinement statistics for HEPC3.1 and HEPC3.4 Fabs structures.

	HEPC3.1	HEPC3.4
Data collection		
Wavelength (Å)	0.98	0.98
Resolution range (Å)	45.87 - 2.79 (2.89 - 2.79)*	79.70 - 3.30 (3.42 - 3.30)
Space group	$I2_13$	R32
Cell, Å (a, b, c)	171.61, 171.61, 171.61	263.79, 263.79, 207.09
Angles (α, β, γ)	90, 90, 90	90, 90, 120
Total reflections	348,624 (27,662)	386,153 (37,296)
Unique reflections	21,030 (2,032)	41,569 (4,124)
Multiplicity	16.6 (13.6)	9.3 (9.0)
Completeness (%)	97.2 (76.6)	99.9 (99.9)
I/σI	8.93 (0.33)	10.09 (0.87)
Wilson B-factor ($Å^2$)	95.23	133.7
R_{merge}	0.22 (10.2)	0.14 (2.86)
R_{pim}	0.05 (2.78)	0.05 (1.01)
$CC_{1/2}$	1.00 (0.22)	1.00 (0.48)
Refinement statistics		
R_{work}	21.16	21.46
R_{free}	24.39	26.04
Number of atoms	3,254	9,368
Protein	3,254	9,368
Ligands		
Solvent		
Protein residues	433	1,239
R.m.s deviations		
Bond lengths (Å)	0.005	0.02
Bond angles (˚)	1.06	1.58
Clashscore	8.4	18.48
Average B-factor	94.69	187.85

*Numbers in parentheses correspond to the highest resolution shell
