# Supplementary material for: Computational identification of HCV neutralizing antibodies with a common HCDR3 disulfide bond motif in the antibody repertoires of infected individuals
Source: Nat Commun. 2022 Jun 8;13:3178. doi: 10.1038/s41467-022-30865-9 (PMC9177688; doi:10.1038/s41467-022-30865-9)
Supplement: Supplementary file 2 — Description of Additional Supplementary Files [file 41467_2022_30865_MOESM2_ESM.docx]

File Name: Supplementary Data 1

Description: Sets of intramolecular distances (CV_respairs.txt), angles (CV_angles.txt), and dihedrals (CV_dihedrals.txt) used for time-lagged independent component analysis (TICA) of the MD simulation trajectories.
