# Supplementary material for: Computational identification of HCV neutralizing antibodies with a common HCDR3 disulfide bond motif in the antibody repertoires of infected individuals
Source: Nat Commun. 2022 Jun 8;13:3178. doi: 10.1038/s41467-022-30865-9 (PMC9177688; doi:10.1038/s41467-022-30865-9)
Supplement: Supplementary file 4 — Reporting Summary [file 41467_2022_30865_MOESM4_ESM.pdf]

nature research

Corresponding author(s): Jens Meiler

Last updated by author(s): May 5, 2022

Reporting Summary

Nature Research wishes to improve the reproducibility of the work that we publish. This form provides structure for consistency and transparency in reporting. For further information on Nature Research policies, see our Editorial Policies and the Editorial Policy Checklist.

Statistics

For all statistical analyses, confirm that the following items are present in the figure legend, table legend, main text, or Methods section.

n/a	Confirmed	
☐	☒	The exact sample size (n) for each experimental group/condition, given as a discrete number and unit of measurement
☐	☒	A statement on whether measurements were taken from distinct samples or whether the same sample was measured repeatedly
☒	☐	The statistical test(s) used AND whether they are one- or two-sided<br>*Only common tests should be described solely by name; describe more complex techniques in the Methods section.*
☒	☐	A description of all covariates tested
☒	☐	A description of any assumptions or corrections, such as tests of normality and adjustment for multiple comparisons
☐	☒	A full description of the statistical parameters including central tendency (e.g. means) or other basic estimates (e.g. regression coefficient) AND variation (e.g. standard deviation) or associated estimates of uncertainty (e.g. confidence intervals)
☒	☐	For null hypothesis testing, the test statistic (e.g. F, t, r) with confidence intervals, effect sizes, degrees of freedom and P value noted<br>*Give P values as exact values whenever suitable.*
☒	☐	For Bayesian analysis, information on the choice of priors and Markov chain Monte Carlo settings
☒	☐	For hierarchical and complex designs, identification of the appropriate level for tests and full reporting of outcomes
☒	☐	Estimates of effect sizes (e.g. Cohen's d, Pearson's r), indicating how they were calculated

Our web collection on statistics for biologists contains articles on many of the points above.

Software and code

Policy information about availability of computer code

Data collection	Next-generation sequencing runs were performed on the Illumina MiSeq platform with a MiSeq Reagent Kit V3 2 x 300 (600 cycle) (MS-102-3003, Illumina).<br>X-ray diffraction data from cryopreserved crystals were collected at the Stanford Synchrotron Radiation Lightsource on beamline 12–2 using a PILATUS 6M detector.<br>Rosetta modeling runs and Molecular dynamics (MD) simulations were performed on ACCRE (The Advanced Computing Center for Research and Education) cluster at Vanderbilt.

Data analysis	Rosetta modeling suit version 3.9 was used to generate and analyze antibody models.<br>For ELISAs binding data and HCV pseudoparticles neutralization assays, analyses of data were performed using Prism version 5 (GraphPad Inc).<br>X-ray diffraction images were processed and scaled using iMosflm and Aimless as implemented in the CCP4 software suite (version 7.0.060). The models were refined and validated using Phenix.refine (version 1.13_2998). Iterative manual model building and corrections were performed using Coot (version 0.9.6). The quality of the final models was examined using MolProbity.<br>Superimposition of the antibody structure models, calculation of the parameters characterizing the overall shape of the Ab HCDR3 loops, and antibody structures-containing figures' rendering was performed using PyMOL (version 2.2.3, Schrodinger, LLC).<br>Amino acid frequency distribution in sequences showed on Figure 1 was analyzed and visualized using WebLogo (https://weblogo.berkeley.edu/logo.cgi).<br>The bioinformatics processing of all NGS data was done using an in-house bioinformatics sequence processing pipeline. An outline of all processing steps is provided in the manuscript.<br>For Molecular dynamics (MD) simulations, systems were parameterized with Leap in AmberTools20. Covalent bonds to hydrogen atoms were constrained with the SHAKE algorithm implemented in Amber20. Simulations were performed with Amber20 using the Particle Mesh Ewald MD CUDA (PMEMD.cuda) application and ff19SB force field. MD simulation trajectory featurization and time-lagged independent component analysis was performed with PyEMMA version 2.5.7.

For manuscripts utilizing custom algorithms or software that are central to the research but not yet described in published literature, software must be made available to editors and reviewers. We strongly encourage code deposition in a community repository (e.g. GitHub). See the Nature Research guidelines for submitting code & software for further information.

Data

Policy information about availability of data

All manuscripts must include a data availability statement. This statement should provide the following information, where applicable:

- Accession codes, unique identifiers, or web links for publicly available datasets
- A list of figures that have associated raw data
- A description of any restrictions on data availability

The crystal structures of the HEPC3.1 and HEPC3.4 Fabs reported in this paper are deposited to the Protein Data Bank under accession numbers 7U0B and 7U0C. Structures deposited by other groups that were used for analysis of the Ab structures with an HCDR3 loop CXXXXC motif in this paper (2FB4, 6C6Z, 5BV7, 4NZU, 3EYF, 6IEA, 5V7U, 6VBQ, 6BKB, 6Q19, 6WO4, 6URH, 4PTU, 6APC, 5IG7, 6BLI, 6Q1J, 6PBV, 6UOE, 6Q1G, 6Q0E, 4XAK, 6MED, 6MEF, 6MEE) are publicly available (www.rcsb.org).

Sequences of the antibodies tested in this study are provided in the manuscript.

NGS data for Ab libraries were deposited to the Sequence Read Archive under accession number PRJNA813433 [https://www.ncbi.nlm.nih.gov/bioproject/PRJNA813433/].

Rosetta Ab models as well as coordinate and restart files for all MD trajectories can be accessed from the GitHub repository [https://github.com/meilerlab/HEPC3].

Field-specific reporting

Please select the one below that is the best fit for your research. If you are not sure, read the appropriate sections before making your selection.

☒ Life sciences ☐ Behavioural & social sciences ☐ Ecological, evolutionary & environmental sciences

For a reference copy of the document with all sections, see nature.com/documents/nr-reporting-summary-flat.pdf

Life sciences study design

All studies must disclose on these points even when the disclosure is negative.

Sample size	No sample-size calculations were performed for the study. Sample sizes were dictated by the overall number of available sequences obtained from the analyzed samples or the nature of the sequences. Only two sibling Abs were found by conventional sequence-based search and both were tested. Ten potentially active Abs were prioritized from the computational survey of the sequence dataset based on their satisfying numerical characteristics and the researcher's manual evaluation.
Data exclusions	No data were excluded from the analysis.
Replication	Two independent experiments were performed for both ELISAs binding and HCV pseudoparticles neutralization assays. Previously characterized antibodies were included in all experiments to verify reproducibility. No unreproducible results were observed and all replication attempts were successful. The results of the binding and neutralization assay which were performed in different laboratories are also in good agreement with each other.<br>Six independent 5.0 µs MD simulations were performed for each of the analyzed Ab. All trajectories were included in the analysis.
Randomization	Ten randomly selected C-X-G-G-X-C HCDR3 motif-containing Abs were obtained from a list of all C-X-G-G-X-C HCDR3 motif-containing Abs except the ones already tested in the study using Bash shuf command.
Blinding	For the first set of the tested antibodies, investigators who performed testing were not aware of the computationally predicted activity/ranking of the Abs being tested. The second pool of the tested antibodies was randomly selected from the Ab libraries, no predictions of their activity were made and, hence, no expectations for the particular outcome could have been made. Results of the computational analysis were assessed by multiple operators to prevent bias.

Reporting for specific materials, systems and methods

We require information from authors about some types of materials, experimental systems and methods used in many studies. Here, indicate whether each material, system or method listed is relevant to your study. If you are not sure if a list item applies to your research, read the appropriate section before selecting a response.

Materials & experimental systems

n/a	Involved in the study
☐	☒ Antibodies
☐	☒ Eukaryotic cell lines
☒	☐ Palaeontology and archaeology
☒	☐ Animals and other organisms
☐	☒ Human research participants
☒	☐ Clinical data
☒	☐ Dual use research of concern

Methods

n/a	Involved in the study
☒	☐ ChIP-seq
☒	☐ Flow cytometry
☒	☐ MRI-based neuroimaging

Antibodies

Antibodies used	Goat anti-human IgG horseradish peroxidase-conjugated secondary antibody (Southern Biotech Cat# 2040-05, , 1:4,000 dilution), ;<br>HCV glycoprotein E2 front layer-specific bNAb HEPC3 (PMID: 31068427), the Ab was previously discovered and characterized by some of the authors of the current research, different dilutions depending on the experiment;<br>HCV glycoprotein E2 front layer-specific bNAb HEPC74 (PMID: 31068427), the Ab was previously discovered and characterized by some of the authors of the current research, different dilutions depending on the experiment;<br>HCV glycoprotein E2 antigenic site 412-binding bNAb HC33.1 (PMID: 27791171), the gene encoding the Ab was synthesized and the Ab was recombinantly expressed and purified for this study by some of the authors of the current research, different dilutions depending on the experiment;<br>New antibodies tested in this paper.
Validation	Goat anti-human IgG horseradish peroxidase-conjugated secondary antibodies are commercially available, have been appropriately validated by manufacturers and this information is provided on their website: https://www.southernbiotech.com/?catno=2040-05&type=Polyclonal#&panel1-1&panel2-1;<br>Activity of all primary antibodies was tested via antigen binding and HCV pseudoparticles neutralization assays. Results for previously characterized Abs were compared with the known activity profiles to confirm their identity. bNAbs HEPC3 and HC33.1 were additionally validated via binding assay using glycoprotein ectodomain E2 variant that contains alanine substitutions that disable binding of HEPC3-like bNAbs (1a157 E2ecto dFRLY).

Eukaryotic cell lines

Policy information about cell lines

Cell line source(s)	HEK293-6E cells were obtained from National Research Council of Canada. Expi293F and Hep3B cells were obtained from American Type Culture Collection (ATCC).
Authentication	None of the cell lines used were authenticated
Mycoplasma contamination	HEK293-6E cells were not regularly tested for mycoplasma contamination. Expi293F and Hep3B cell lines tested negative for mycoplasma contamination.
Commonly misidentified lines<br>(See ICLAC register)	None

Human research participants

Policy information about studies involving human research participants

Population characteristics	Previously banked peripheral blood mononuclear cells from two hepatitis C virus infected individuals were provided as de-identified, identity-unlinked samples from the Baltimore Before and After Acute Study of Hepatitis (BBAASH) cohort at the Johns Hopkins University School of Medicine.
Recruitment	No subjects were recruited for this study. Human blood samples used had been previously banked as part of the BBAASH cohort at the Johns Hopkins University School of Medicine. Participants in the BBAASH cohort are volunteers who inject drugs and are therefore at higher risk of hepatitis C virus infection. Participants sign written informed consent to participate in the cohort.
Ethics oversight	This study was approved by the Institutional Review Board of the Johns Hopkins University School of Medicine.

Note that full information on the approval of the study protocol must also be provided in the manuscript.
